# The prevalence of autism spectrum traits and autism spectrum disorders in children and adolescents with obsessive compulsive disorder: systematic review and meta-analysis

**DOI:** 10.1192/bjo.2025.10936

**Published:** 2026-01-16

**Authors:** Claire Tiley, Paraskevi Lampropoulou, Myrto Samara, Marinos Kyriakopoulos

**Affiliations:** Child and Adolescent Mental Heath Services, South London and Maudsley NHS Foundation Trust, London, UK; Child and Adolescent Eating Disorders Service, Oxford Health NHS Foundation Trust, London, UK; Department of Psychiatry, Faculty of Medicine, University of Thessaly, Larissa, Greece; Department of Psychiatry and Psychotherapy, Klinikum rechts der Isar, School of Medicine, Technical University of Munich, Munich, Germany; Department of Psychiatry, National and Kapodistrian University of Athens, Athens, Greece; Department of Child and Adolescent Psychiatry, Institute of Psychiatry, Psychology & Neuroscience, King’s College London, London, UK

**Keywords:** Obsessive Compulsive Disorder, Autism Spectrum Disorder, children, adolescents, co-morbidity

## Abstract

**Background:**

Autism spectrum disorder (ASD) and obsessive–compulsive disorder (OCD) may coexist in children and adolescents and present with several overlapping features.

**Aims:**

We aimed to assess the prevalence of ASD traits and diagnosis in children and adolescents with OCD, explore the correlation between OCD severity and ASD traits/diagnosis, and examine the impact of ASD traits/diagnosis on global functioning in this population.

**Method:**

Electronic searches were carried out on Pubmed, Embase and PsycINFO, using selected keywords and specified inclusion and exclusion criteria. Meta-analysis was performed with R Version 4.3.1.

**Results:**

Of 1410 studies initially identified, 29 reported on the prevalence of ASD traits or diagnosis. Pooled mean prevalence rate was 8.0% (95% CI 5.0–13%). ASD questionnaire scores were higher in OCD versus control groups (standardised mean difference: 1.23; 95% CI 0.76–1.69). There was limited significant correlation between ASD questionnaire scores and OCD questionnaire scores, and no significant differences in these scores were demonstrated between OCD samples and samples diagnosed with comorbid OCD and ASD (mean difference −0.41; 95% CI −1.23 to 0.40). Functional impairment appeared elevated with ASD traits/diagnosis in OCD, but meta-analysis feasibility was limited.

**Conclusions:**

This review indicates higher ASD traits and diagnosis in children and adolescents with OCD compared with the general population. Limited data and methodological constraints in trials limit generalisability, warranting further research.

Obsessive–compulsive disorder (OCD) is a psychiatric disorder characterised by recurrent intrusive thoughts, impulses and images and/or repetitive rituals or mental acts, which affects between 2 and 4% of children and adolescents.^1–3^ OCD has a number of similarities to autism spectrum disorder (ASD), a neurodevelopmental disorder characterised by impaired communication, impaired reciprocal social interaction and restricted and repetitive interests or patterns of behaviour, which affects more than 2.7% of the general child and adolescent population.^4,5^ The phenotypical similarities between the two disorders include repetitive and stereotypical behaviours, the need for sameness and inflexibility.^6^ Touching, tapping, ordering and hoarding are all repetitive behaviours found in both ASD and OCD.^7–10^ Although relatively similar, the repetitive behaviours serve different functions in these two conditions.^11–13^ In OCD, typically the behaviour is carried out to relieve anxiety, is egodystonic and overall is associated with distress and anxiety, whereas in ASD, the behaviour is not usually associated with distress, but rather intense preoccupations with new objects or idiosyncratic circumscribed interests.^11–13^ The overlap between the two disorders led to the nosological debate as to whether OCD had been correctly placed as an anxiety disorder in the DSM-IV-TR, and, in more recent classifications, OCD has been moved under the Obsessive Compulsive and Related Disorders category’.^14,15^

Research has suggested shared underlying mechanisms between OCD and ASD.^16–18^ In both ASD and OCD, neuroanatomical findings indicate that thinner cortical regions are associated with an increase in social deficits.^19^ This is supported by previous studies that found reduced temporal/parietal cortical thickness in patients diagnosed with ASD or OCD.^20,21^ Abnormalities with the prefrontal cortex have also been identified in both OCD and ASD.^22–24^ Genetic factors further emphasise the association between the two disorders, with some studies having found that family members of individuals with ASD are more likely to demonstrate compulsive personality traits.^25^ A systematic review and meta-analysis found that 10% of parents of children with ASD had a diagnosis of OCD, which was elevated compared with OCD prevalence in the general population.^26–28^ Ozyurt and colleagues also found that mothers of children and adolescents with OCD scored significantly higher on the Autism Quotient compared with mothers of the control group, suggesting further overlap between these conditions.^29^

OCD and ASD commonly co-occur in children and adolescents. A systematic review and meta-analysis completed by Aymerich and colleagues in 2024 identified a pooled prevalence rate of ASD in children, adolescents and young adults with OCD of 9.46%.^30^ To the authors’ knowledge, no systematic review and meta-analysis to date has evaluated both ASD diagnosis and traits within a population of children and adolescents with OCD.^30^

Functional impairment is associated with both ASD and OCD individually in the paediatric population, but there has been less exploration of the impact on functioning when both conditions co-exist. Comorbid OCD and ASD is associated with an increase in functional impairment in school, at home and in social aspects of life when compared with children and adolescents with OCD only.^30,31^ The additive effects of comorbidities may be associated with an increase in functional impairment.^32^ To date, the impact of comorbid ASD or ASD traits on global functioning in children and adolescents with OCD has not been adequately explored.

The aims of this systematic review and meta-analysis are:

- To provide a prevalence estimate of ASD traits and diagnosis in children and adolescents with a diagnosis of OCD.
- To compare ASD trait severity between child and adolescent OCD populations and control groups/normative data.
- To examine whether the severity of OCD symptoms is related to the severity of ASD traits or presence of a diagnosis in children and adolescents diagnosed with OCD.
- To examine whether the severity of comorbid ASD traits or presence of a diagnosis in children and adolescents diagnosed with OCD impact negatively on their global functioning.

## Method

This systematic review was registered with PROSPERO before data collection (number: CRD42018106411), and details can be found in Supplementary Fig. 1 available at https://doi.org/10.1192/bjo.2025.10936.

### Search strategy

PRISMA guidelines were followed throughout this systematic review.^33^ Electronic searches were carried out on 25 February 2025, using Embase, Medline and PsycINFO, with the keywords of ‘obsessive compulsive disorder,*’ ‘Obsessive-Compulsive Disorder/’ ‘ocd,’ ‘autis,*’ ‘Asperger,*’ ‘ASD,’ ‘child,*’ ‘adoles,*’ ‘p?diatric,’ ‘youth,’ ‘juvenile,’ ‘teen,*’ ‘infant,’ ‘young people’ and ‘young person.’ Thesaurus searches were also used for the main keywords, including ‘adolescent’, ‘ASD’ and ‘obsessive-compulsive disorder’. Boolean operators OR and AND were used as appropriate. The language for the searches was restricted to English. The publication type was restricted to journal article, but no restrictions were set for the date of publication. The search was also restricted to human studies. The author (C.T.) conducted a systematic search of the literature alongside a medical librarian. Screening of titles, abstracts and full text was performed by two authors (C.T. and P.L.) working independently and in duplicate. Interrater agreement was calculated with Cohen’s *κ* coefficient. Any discrepancies were resolved through discussion between the two reviewers until a consensus was reached, or by consultation with a third senior author (M.K.) when necessary. The search was completed by manually searching through previous literature reviews, systematic reviews and meta-analysis on this topic.

### Eligibility criteria

For this review, the participants had to be up to 18 years of age and have a diagnosis of OCD (either according to the ICD or the DSM). Papers that evaluated (a) ASD or associated traits in young people with a diagnosis of OCD, (b) the relationship between OCD severity and ASD trait severity/diagnosis or (c) the relationship between ASD trait severity/diagnosis and global functioning in children and adolescents diagnosed with OCD, were included in this review. A diagnosis of pervasive developmental disorder was also included as falling under the umbrella of ASD. Any study design where this data could be extracted or calculated were included. Papers investigating disorders related to OCD, including body dysmorphic disorder, compulsive skin picking, trichotillomania and hoarding disorder, were excluded. In addition, papers were excluded if the sample included adults where data about the adolescent sample could not be extracted and where this data was not available from the authors. Papers that were not written in English, poster abstracts and dissertations were also excluded. If there was a potential for sample overlap between two or more studies, where original authors did not respond, author C.T. presumed sample overlap between the studies and excluded the study with the smaller sample size.

### Strategy for data extraction, synthesis and statistics

Data extraction commenced on 28 May 2025. The authors (C.T. and P.L.), working independently and in duplicate, extracted data from the identified studies. Interrater agreement was calculated with Cohen’s *κ* coefficient. Any discrepancies were resolved through consensus and by consulting a senior author (M.K.). Where specific data was not available from the manuscript, author C.T. contacted authors of the original studies to request the required data. Authors P.L. and M.S. completed the methods of data synthesis and statistics described below.

#### Prevalence of ASD diagnosis

Prevalence of ASD diagnosis was reported as a percentage, calculated by using the total number of children and adolescents diagnosed with comorbid ASD as the numerator and the total OCD sample as the denominator.

In regards to the meta-analysis, for the summary effect of the ASD diagnosis prevalence, unadjusted prevalence rates were calculated based on the information of crude numerators and denominators provided by individual studies. Prevalence was reported with 95% confidence intervals and the degree of heterogeneity was estimated by *I* ^2^-statistics.^34^ As considerable heterogeneity was expected, the random effects model was primarily employed, but an estimate based on common effects model was also presented. Meta-analytic calculations were performed with R version 4.3.1 on macOS.

We aimed to assess publication bias with funnel plots and Egger’s regression test,^35^ provided that a reasonable number of studies were available, defining this minimum as ten studies.^34^

#### Prevalence of ASD traits

ASD traits were reported as the percentage of participants scoring over a specified cut-off score in the questionnaires used to screen for or assess ASD. Questionnaires used to measure ASD traits included the Social Communication Questionnaire (SCQ), Social Responsiveness Scale (SRS), Social Responsiveness Scale – Version 2 (SRS-2), Autism Spectrum Screening Questionnaire (ASSQ), Autism Quotient and Children’s Social Behaviour Questionnaire (CSBQ).^36–42^

#### Comparison of ASD traits between OCD samples and controls

Aggregate mean scores of the relevant ASD questionnaires were compared between the OCD group and the control group (those individuals without a diagnosis of OCD).

#### Exploring the effect of comorbid ASD diagnosis on OCD severity

Aggregate mean scores of the Children’s Yale–Brown Obsessive–Compulsive Scale (CY-BOCS) in an OCD-only group were compared with comorbid group diagnosed with OCD and ASD.^43,44^

For comparisons above, a pairwise meta-analysis was conducted to provide a more comprehensive description and visualisation of score differences between groups in the questionnaires used to measure ASD traits or OCD symptoms. The effect size used was the mean difference. However, if included studies used different scales, the standardised mean difference (SMD), expressed as Hedges’ adjusted *g*-statistic, was estimated. The pooling of studies utilised the standard inverse variance weighting method. Considering the anticipated heterogeneity among the studies, which was quantified by the *I* ^2^-statistic,^34^ the Der-Simonian and Laird random effects model was consistently employed.^43,44^ Meta-analytic calculations were performed with R Version 4.3.1 on macOS.

#### Reviewing the effect of comorbid ASD trait severity on OCD severity

Correlation of aggregate mean scores of the relevant questionnaires used to measure ASD and aggregated mean scores of the CY-BOCS questionnaire (used to measure OCD) were described.^43,44^

#### Reviewing the effect comorbid ASD trait severity on functional impairment

Correlation of aggregate mean scores of relevant ASD questionnaires and questionnaires used to measure functional impairment (Children’s Global Assessment Scale (CGAS) score and Child OCD Impact Scale – Parent Version (COIS-P))^45,46^ were described. More details on the questionnaires used for ASD, OCD and functional impairment can be found in Supplementary Table 2.

#### Analysing the effect of comorbid ASD diagnosis on functional impairment

Aggregate mean scores of questionnaires used to measure functional impairment were compared between an OCD-only group and a comorbid group diagnosed with OCD and ASD.

### Quality assessment

To assess quality of the studies, the Newcastle–Ottawa Scale adaptions for case–control, cohort and cross-sectional studies were used.^47,48^ To assess the quality of any randomised control trials, the Jadad score was used.^49^ The Cochrane Risk of Bias tool was not used as this paper was not examining interventions or treatments. Quality was assessed independently and in duplicate by two authors (C.T. and P.L.), with interrater agreement calculated with Cohen’s *κ* coefficient. Any discrepancies were resolved through discussion between the two reviewers (C.T. and P.L.) and, when necessary, consultation with a senior author (M.K.).

## Results

### Identified studies

**Fig. 1:**
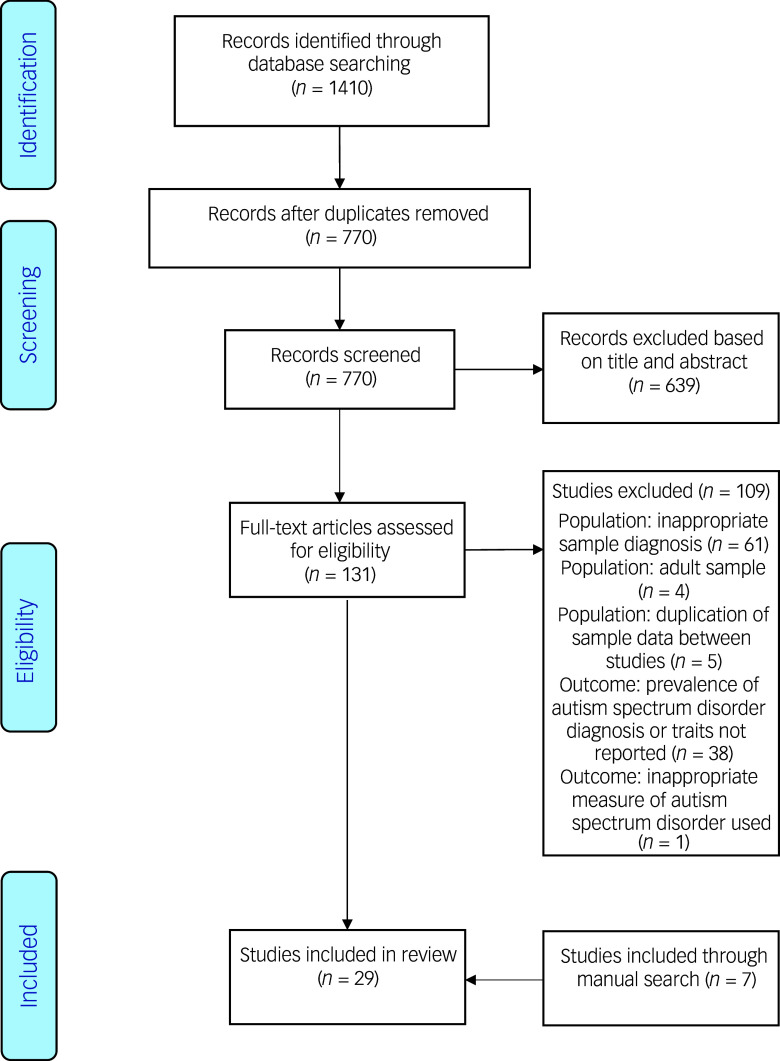
Journal identification process.

A total of 1410 studies were identified through the electronic search, and 770 studies remained once duplicates were removed. The remaining studies were screened based on their title and abstract. After screening 131 full-text studies, we excluded 109 studies (as shown in Fig. 1), including five studies displaying potential sample duplication.^11,18,19,50,51^ This left 22 studies for inclusion. Seven additional studies were included through manual searches, meaning a total of 29 studies were included in the review (Fig. 1 and Supplementary Table 3).^6,8,12,31,50,52–76^ Cohen’s *κ* coefficient indicated near perfect agreement for screening (*κ* = 0.95) and there were no conflicts in data extraction. From the 29 studies where data extraction was possible, 13 were case–control studies, ten cross-sectional studies, four cohort studies and two randomised controlled trials (full details available in Supplementary Table 3).

Of the 29 studies included in this review, 13 studies reported on the prevalence of ASD diagnosis in children and adolescents diagnosed with OCD, eight studies reported on the prevalence of ASD traits in this population scoring above a specified clinical cut-off, and seven studies compared ASD measure scores in an OCD sample versus a control group or normative data.

The SCQ was the most commonly administered questionnaire used to measure ASD traits or to support an ASD diagnosis, followed by the ASSQ. Five studies used the SCQ^6,12,29,63,75^ and four studies used the ASSQ,^8,52,74,77^ three studies used the SRS,^6,53,56^ one of which used the second edition of the SRS (SRS-2).^53^ One study used both the SCQ and SRS.^6^ Two studies used the Autism Quotient^58,76^ and one study used the CSBQ.^57^

#### Prevalence of ASD diagnosis

**Fig. 2:**
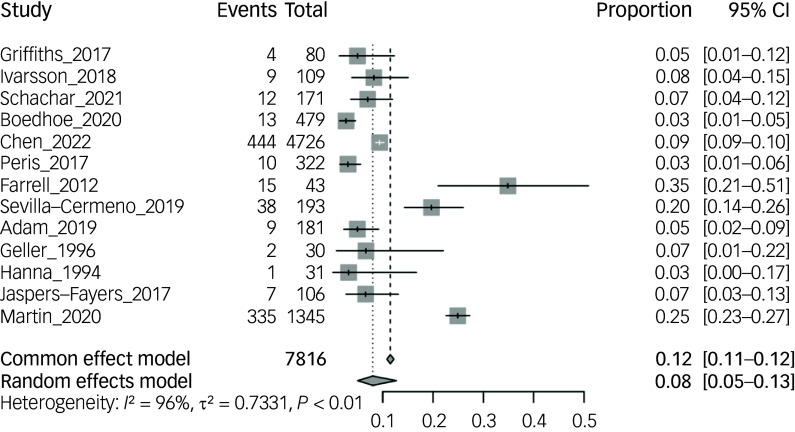
Prevalence of autism spectrum disorder diagnosis in children and adolescent obsessive–compulsive disorder samples.

In the 13 studies that measured the prevalence of ASD diagnosis in 7816 children and adolescents with OCD (51.8% male), prevalence rates ranged from 2.71 to 34.9%.^8,53,59,60,65–72,78^ The pooled mean prevalence, using a random effects model, was 8.0% (95% CI 5.0–13.0%, *P* < 0.01). As displayed in Fig. 2, three of the studies appear to be outliers compared with the ten other studies, which may be explained by sample variation. There were not enough data to examine gender difference specific to ASD prevalence in this OCD population; however, four studies reported either increased prevalence rates within their male OCD population or a higher proportion of males in those diagnosed with ASD (Supplementary Table 3).^59,60,68,71^

#### Prevalence of significant ASD traits

Eight studies reported on the prevalence of significant ASD traits as per clinical cut-off scores on the SCQ, SRS, ASSQ and CSBQ. Different definitions of trait cut-offs and scales were used across the studies, making it challenging to summarise and review the data both qualitatively and quantitatively. Prevalence rates are illustrated in Table 1.

**Table 1:** Prevalence rates of significant autism spectrum disorder traits in children and adolescents diagnosed with obsessive–compulsive disorder (OCD)

Study	Questionnaire measure	Specified cut-off score	Prevalence rate of sample scoring equal to or above specified cut-off (%)	Gender differences
Arildskov et al 2016^52^	ASSQ	≥13 ≥17	17.1 9.7	25.6% of the male OCD sample scored ≥13 on ASSQ compared with 9.1% of female OCD sample
Griffiths et al 2017^53^	SRS-2	>60	57.5	Not reported on
Hojgaard et al 2023^77^	ASSQ	≥17	9.7	In the higher autism spectrum disorder trait group, 72% of the sample were male compared with the OCD-only group, where 46.1% were male
Mahjoob et al 2024^75^	SCQ	≥11	13	Not reported on
Stewart et al 2016^6^	SRS SCQ	>60 ≥15	36.2 2.4	Not reported on
Sturm et al 2018^56^	SRS	>60	14.29	Not reported on
Weidle et al 2012^12^	SCQ SCQ	≥15 ≥9	0.95 21	Not reported on
Wolters et al 2016^57^	CSBQ	≥95th percentile	27.6	Not reported on

ASSQ, Autism Spectrum Screening Questionnaire; SRS-2, Social Responsiveness Scale - Version 2; SCQ, Social Communication Questionnaire; SRS, Social Responsiveness Scale; CSBQ, Children’s Social Behaviour Questionnaire.

#### Comparisons of ASD questionnaire scores between OCD group and controls

**Fig. 3:**
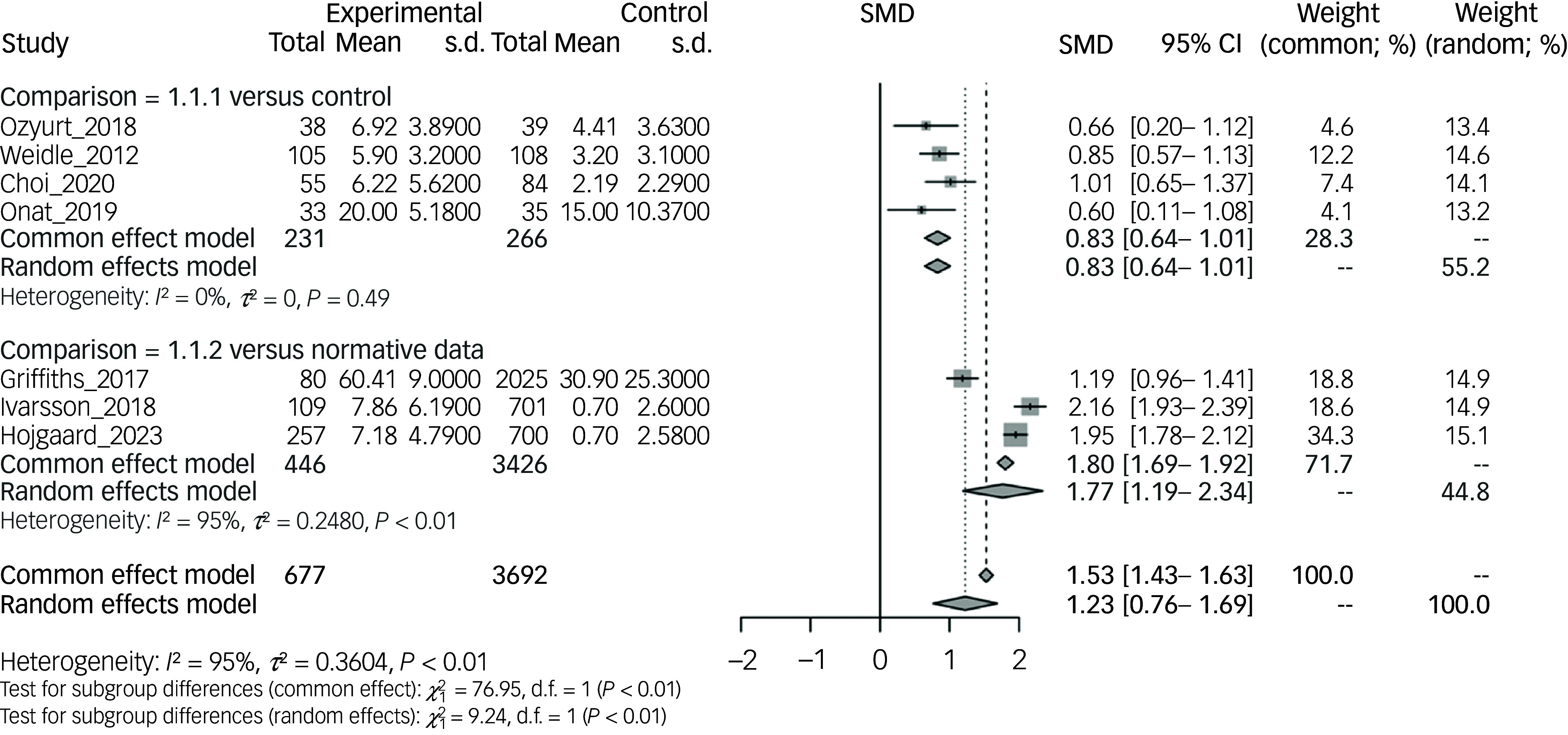
Comparison of autism spectrum disorder measure aggregate mean scores of obsessive–compulsive disorder samples versus control samples or normative data. SMD, standardised mean difference.

In eight of the 29 studies, data from an OCD group were compared with a normotypical control group or normative data.^8,12,29,53,63,74,76,77^ Only seven of the studies could be included in the meta-analysis, as the s.d. for mean scores in one study were not available.^74^ In all studies, participants with an OCD diagnosis had higher scores in the scale measuring ASD symptom severity (overall SMD of 1.23, 95% CI 0.76–1.69) (Fig. 3).

Four studies compared an OCD group with a control group consisting of normotypical children and adolescents. These studies included a total of 266 participants; ASD symptom severity was measured with the SCQ questionnaire in three studies and the Autism Quotient in one study. Control groups had significantly lower ASD symptom severity when compared with OCD groups, with an overall SMD of 0.83 (95% CI 0.64–1.01), as displayed on the forest plot in Fig. 3 (Comparison: 1.1.1 versus control).

As for the comparison with normative data, three studies including a total of 446 participants used either the SRS-2 or ASSQ questionnaires as a measure of ASD symptom severity. Normative data reported significantly lower ASD symptom severity compared with the OCD groups (SMD 1.77, 95% CI 1.19–2.34) as illustrated in Fig. 3 (Comparison: 1.1.2. versus normative data). There was not enough data to examine generalised gender differences; however, Ivarsson and colleagues reported that males obtained statistically significant higher total SCQ scores compared with females (*P* = 0.006).^8^

### Correlation between OCD severity and the severity of ASD traits

Nine of the studies included in the review reported on the correlation between OCD severity and the severity of ASD traits (Table 2).^6,8,29,52,53,55,57,73,76^ ASD traits and OCD severity were measured by questionnaire scores. ASD traits were measured by the SCQ, SRS, ASSQ, Autism Quotient and CBSQ. OCD severity was measured with the CY-BOCS. More details on the questionnaires used can be found in Supplementary Table 2.

**Table 2:** Correlation between measures of obsessive–compulsive disorder (OCD) and autism spectrum disorder in children and young people with a diagnosis of OCD

Study	Questionnaire measures	Correlation	Significance	Additional information
Arildskov et al (2016)^52^	ASSQ total and CY-BOCS	Three-step multiple regression analysis F(10,242) = 2300,	Yes *P* = 0.014	Motor/tic/OCD subscale was the only variable that significantly contributed
Griffiths et al (2017)^31^	Total SRS and total CY-BOCS	Pearson’s correlation *r* = 0.13	No *P* = 0.16	–
Ivarrson et al (2008)^8^	Total ASSQ and total CY-BOCS	Spearman’s correlation *r* = 0.02	No	–
Memis et al (2019)^55^	Autism Quotient: Communication subscale versus total obsession score	Spearman’s correlation *r* = 0.48	Yes *P* = 0.007	No significant total correlation between total Autism Quotient score and CY-BOCS
	Autism Quotient: Communication subscale versus total compulsion score	Spearman’s correlation *r* = 0.40	Yes *P* = 0.02	
Ozyurt et al (2018)^29^	Total SCQ and total CY-BOCS	Spearman’s correlation *r* = 0.08	No *P* = 0.632	–
Onat et al (2019)^76^	Total Autism Quotient and CY-BOCS	Spearman’s correlation *r* = 0.42	Yes *P* < 0.05	Significant moderate correlation between total Autism Quotient scores and CY-BOCS
Salemink et al (2023)^73^	Total SRS and total CY-BOCS	Spearman’s correlation *r* = 0.087	No *P* = 0.064	–
Stewart et al (2016)^6^	SRS total versus CY-BOCS total	Pearson’s *r* = 0.21	Yes *P* < 0.05	Significant correlation between total SCQ and SRS scores
	SCQ total versus CY-BOCS total	Pearson’s *r* = 0.11	No *P* > 0.05	
Wolters et al (2016)^57^	CBSQ total versus CY-BOCS total	Linear regression *r* = 0.35	Yes *P* = 0.009	–

ASSQ, Autism Spectrum Screening Questionnaire; CY-BOCS, The Children’s Yale Brown Obsessive Compulsive Scale; SRS, Social Responsiveness Scale; SCQ, Social Communication Questionnaire; CBSQ, Children’s Social Behaviour Questionnaire.

Four of the studies found that there was no significant correlation between questionnaire scores used to measure the severity of OCD symptoms and measures assessing ASD traits.^8,29,53,73^

Wolters and colleagues found a weak correlation between mean CY-BOCS scores and the CSBQ scores (*r* = 0.35, *P* = 0.009).^57^ Stewart and colleagues found a weaker correlation between mean CY-BOCS scores and mean total SRS scores (*r* = 0.21, *P* < 0.05), and between mean CY-BOCS score and the autistic mannerisms subscale of the SRS (*r* = 0.21, *P* < 0.05). The correlation between mean CY-BOCS and SCQ score was not significant.^6^

Onat and colleagues found a moderate correlation between mean CY-BOCS scores and Autism Quotient scores (*r* = 0.42, *P* < 0.05).^76^ Arildskov and colleagues also found a significant correlation between mean CY-BOCS scores and ASSQ. However, the motor/tic/OCD subscale was the only variable that significantly contributed to this. The autistic style and social difficulties subscales did not contribute significantly, as these autism specific symptoms did not significantly correlate with the mean CY-BOCS score.^52^

In the 2019 study by Memis and colleagues, there was no significant correlation found between mean CYBOCS score and mean total Autism Quotient score; however, the study did find that that there was a significant correlation between the mean total compulsion score of the CY-BOCS score and the communication subscale of the Autism Quotient (*r* = 0.40, *P* = 0.02).^55^

### The impact of ASD diagnosis on the severity of OCD

**Fig. 4:**
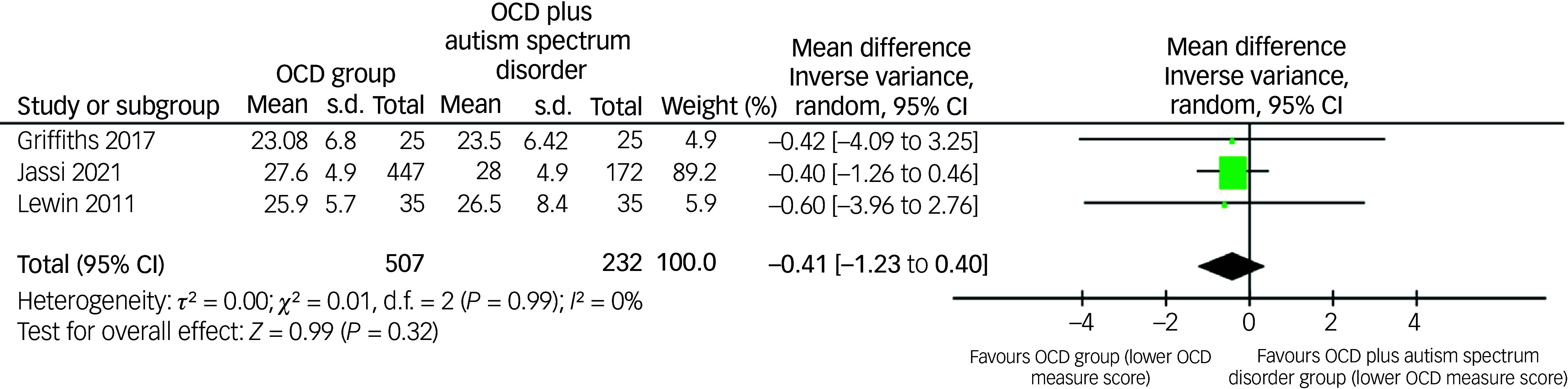
Comparison of obsessive–compulsive disorder (OCD) measure aggregate mean scores of OCD samples and comorbid OCD and autism spectrum disorder samples.

Three studies reported on the impact of ASD diagnosis on the severity of OCD by comparing mean OCD scores in an OCD-only group to an OCD group with comorbid ASD.^6,31,62^ The CY-BOCS was used as a measure of OCD severity in each study (Fig. 4). No difference was found between the two groups (mean difference: −0.41; 95% CI −1.23 to 0.40) (Fig. 4).

### The impact of ASD trait severity or diagnosis on the global functioning of OCD samples

Three studies reported on the impact of significant ASD traits or diagnosis on the global functioning of individuals with OCD. Two studies used the COIS-P to measure the impact of functioning and one study used the CGAS score.^31,53,59^

Griffiths and colleagues found that the mean COIS-P impact score was weakly correlated with the mean SRS total *t*-score (*r* = 0.23, *P* = 0.02). The study reported that SRS total t-score was a significant predictor of OCD-related functional impairment.^53^

Griffiths and colleagues also found that when comparing total psychosocial impact on functioning between a comorbid OCD and ASD group and an OCD-only group, independent group *t*-tests showed significant differences between the groups *t* ^35^ = 3.41, *P* = 0.002, Cohen’s *d* = 1.02), demonstrating a large effect size. This indicated that there was a greater impairment in functioning in the comorbid group. Significant differences between the groups were also demonstrated when comparing specific aspects of the COIS-P, including school functioning, social functioning and home and family activities (Table 3).^31^ Martin and colleagues also reported that participants with comorbid OCD and ASD presented with greater functional impairment when measured by the CGAS score (mean: 44.30) compared with the OCD-only group (mean: 49.06, Cohen’s *d* = 0.42, *P* < 0.001), demonstrating a small to medium effect size (Table 3).^59^ As only two studies directly compared aggregate mean questionnaire scores between groups, meta-analysis was not performed.

**Table 3:** Comparison of global functioning questionnaire scores between obsessive–compulsive disorder (OCD) only and comorbid obsessive–compulsive disorder and autism spectrum disorder groups

Study	Questionnaire measure	OCD-only mean score	OCD and autism spectrum disorder: mean score	Effect measure
Griffiths et al 2017^53^	COIS-P	Mean: 31.87, s.d. 14.87, *n* = 25	Mean: 51.82, s.d. 23.26, *n* = 25	Cohen’s *d* = 1.02 Large effect size *P* = 0.002
Martin et al 2020^59^	CGAS	Mean: 49.06, s.d. 15.57, *n* = 1010	Mean: 44.30, s.d. 3.29, *n* = 335	Cohen’s *d* = −0.42 Small-medium effect size *P* < 0.001

COIS-P, Child Obsessive-Compulsive Impact Scale (parent version); CGAS, Children’s Global Assessment Scale.

### Quality assessment

There was variation in the study designs included within this systematic review and meta-analysis. The majority of studies used a case–control design, three studies used a cross-sectional design, cross-sectional data was also taken from a randomised controlled trial in one study and three studies were cohort studies. The number of randomised controlled trials was not important as the current systematic review and meta-analysis was not examining an intervention. Cohen’s *κ* coefficients indicated near perfect agreement for quality assessment (*κ* = 0.94). The quality assessment of each study is detailed in Supplementary Table 4.

Overall, the quality of studies included was good, particularly for the comparability, exposure and outcome domains for each study (Supplementary Table 4). All of the case–control studies lacked information on non-responders in both case and control groups and did not report whether the characteristics of non-responders matched those of participants included in the study, which may have contributed to non-response bias. Six studies had particularly small sample sizes, which may limit generalisability.^56,58,67,68,74,76^ None of the cross-sectional studies used sample size calculations.

## Discussion

### Prevalence of ASD diagnosis and traits in OCD

Our systematic review and meta-analysis, to the best of our knowledge, is the first to report on the prevalence of both ASD diagnosis and traits in children and adolescents up to 18 years of age with a diagnosis of OCD, as well as comparing trait severity between this OCD sample and control samples. We identified 13 studies reporting on the prevalence of ASD diagnosis within this pooled OCD sample of 7816 patients, the largest pooled sample to date, finding a pooled prevalence rate of 8.0% (95% CI 5.0–13.0%, *P* < 0.01). The findings of this study also suggested an increase in prevalence of ASD traits in children and adolescents with OCD when compared with the general paediatric population.

Our review supports other previous suggestions of comorbidity between the two diagnoses.^30,79^ Aymerich and colleagues have previously completed a systematic review and meta-analysis on the prevalence of ASD diagnosis in a population of children and young people with OCD, reporting a pooled prevalence rate of 9.46%. The elevated prevalence of ASD traits and diagnosis within the OCD population may be explained by shared underlying mechanisms of both disorders.^16–18^ Both neuroanatomical findings and genetic studies support this finding, including a systematic review and meta-analysis identifying that 10% of parents of children with ASD had a diagnosis of OCD,^27^ which is elevated compared with the prevalence of OCD within the general population.^17,18,20,26,27^

In our review, ASD prevalence rates were similar between studies, ranging from 2.71 to 9.39% in most studies. Three studies found higher prevalence rates of 19.7, 24.9 and 34.9%, which are outliers compared with the other ten studies.^59,66,72^ This may be explained by differences in the representativeness of the samples within these studies. Martin and colleagues reviewed records from children and adolescents who presented to South London and Maudsley NHS Foundation Trust who had a diagnosis of OCD and/or ASD. The OCD service provided by South London and Maudsley NHS Foundation Trust is a national and specialist OCD service that assesses and treats children and adolescents who have not responded to standard treatment for OCD by community child and adolescent mental health services in England. It is possible that the prevalence rates were higher in this sample because having comorbid ASD would likely impact the treatment response of these individuals, meaning they required referral to this specialist service.^59^ Sevilla-Cermeno and colleagues reviewed the records of all individuals living in Sweden between a specified time period, which may be more representative of the general community than other studies identified that reported on clinical samples.^72^

ASD trait prevalence was not included in the meta-analysis because of the heterogeneity between the questionnaire measures used to determine the percentage of participants scoring over clinical-cut-off for ASD traits. Particularly regarding the ASSQ and SRS-2, two different clinical cut-offs were used to determine whether moderate or severe traits were present. Significant differences were also seen in prevalence rates of significant ASD traits above the related cut-off, depending on the questionnaire measure used. Prevalence rates of studies using the SCQ found prevalence rates of between 1.0 and 14% compared with those using the ASSQ, SRS-2 and CSBQ, where rates ranged from 14.3 to 48.0%. This may be explained by the differences in sensitivity and specificity between the questionnaires. Regarding the SCQ, a meta-analysis by Chestnut and colleagues found that differences in sampling affected the accuracy of the SCQ as a screening tool.^61^ Particularly, clinic-referred samples have been found to perform poorer in classifying those with ASD, as was the case with many of the samples included in our systematic review.^80^ Other research has suggested that reducing the cut-off to 11 rather than 15 as being most accurate in children aged 4–12 years, with Stewart and colleagues questioning whether the yes/no responses in the SCQ were able to identify the milder spectrum of ASD traits.^6,81,82^ Conversely, both studies by Griffiths and colleagues and Stewart and colleagues raised concerns as to whether the SRS is too sensitive a measure to differentiate OCD from ASD, and is less effective in assessing the function of the behaviour, but rather captures symptom overlap.^6,53^ All these considerations may be related to the large discrepancy in the prevalence of significant ASD traits in the OCD samples between questionnaires.

### The impact of ASD trait severity or diagnosis on the severity of OCD

Our review explored whether there was an association between the severity of OCD and ASD traits within the child and adolescent OCD population. Only three out of the nine studies that reported on this found that there was a significant correlation between the two when focusing on total questionnaire scores (Table 2).^52,57,76^ Meta-analysis also supported this lack of evidence when comparing OCD scores in an OCD only group with a comorbid OCD and ASD group, but only three studies provided data (Fig. 4). Amongst the studies, Arildskov and colleagues commented that the motor/tic/OCD subscales of the ASSQ were the only variables that significantly correlated with OCD severity as measured by CY-BOCS.^52^ This supports comments made in some studies as to whether the questionnaire measures used could successfully differentiate between the repetitive and stereotypical behaviours seen in ASD versus the repetitive ritualistic behaviours present in OCD.^29,53^ In contrast, a correlation between only the communication subscale of the Autism Quotient measure and both lifetime obsessions and compulsions were reported, suggesting correlation is not only related to the subscales associated with repetitive behaviour.^55^ Again possible limitations of the SRS questionnaire to assess the function of the behaviour measured or to capture the symptom overlap between the two conditions need to be considered.^6^

### The impact of ASD trait severity or diagnosis on global functioning in OCD

Although there were not enough studies to perform a meta-analysis, our review suggests that increased ASD traits or diagnosis in children and adolescents with OCD are associated with an increase in functional impairment across school, home and social settings.^31,53,59^ This is also supported by Aymerich and colleagues review who concluded that there were high rates of functional impairment in comorbid diagnosis of ASD and OCD, but were not able to use meta-analysis to support this.^30^ As assessment of functioning is important for both the diagnosis and monitoring of OCD, further research in this area would be of significant benefit.

### Limitations

Limitations highlighted in studies of the current systematic review and meta-analysis included the reliance on parental reporting in the measures used, which may skew the data. The lack of information on responders versus non-responders in the case-control studies may have contributed to non-response bias limiting the generalisability of our findings. Psychometrically validated interviews were suggested as a more reliable measure for identifying the diagnosis or traits of ASD. Differences in sample sizes was also noted in studies as a limitation. It was particularly evident when using the ASSQ normative data, which was based on a sample number of 1401, much larger than the OCD samples being studied.^8,52^ Smaller samples used may not be generalisable to the general population, and studies with stricter exclusion criteria may not be generalisable to youth with more complex symptom presentation. Some children and adolescent in the OCD groups were also taking pharmacological treatment, which may affect findings specifically related to the correlation between the severity of OCD and ASD traits.

### Clinical implications

This systematic review and meta-analysis suggests an increase in prevalence of ASD diagnosis and traits in those individuals with OCD, with comorbidity contributing to more severe functional impairment and treatment resistance to standard care. Comorbid cases may require different treatment approaches or modifications of usual interventions.^83,84^ By screening for and identifying those with co-occuring OCD and ASD, the rates of treatment resistance may decline and length of illness and severity could reduce.

In conclusion, this systematic review and meta-analysis suggests that there is an increased prevalence of ASD traits and diagnosis in children and adolescents with OCD. However, the trials have inherent methodological limitations that restrict the generalisability of these findings. On reviewing the association between OCD severity and ASD trait severity or the presence of a diagnosis, there was conflicting evidence and further research in this area is needed. Finally, this review supports that ASD trait severity or the presence of a diagnosis is associated with an increase in functional impairment, including school functioning, social functioning and both home and family activities; however, data was limited and additional research is warranted.

## Supporting information

Tiley et al. supplementary material 1 Tiley et al. supplementary material

Tiley et al. supplementary material 2 Tiley et al. supplementary material

Tiley et al. supplementary material 3 Tiley et al. supplementary material

## Data Availability

Data availability is not applicable to this article as no new data were created or analysed in this study.

## Supplementary material

The supplementary material is available online at https://doi.org/10.1192/bjo.2025.10936
