# Supplementary material for: The prevalence of autism spectrum traits and autism spectrum disorders in children and adolescents with obsessive compulsive disorder: systematic review and meta-analysis
Source: BJPsych Open. 2026 Jan 16;12(1):e39. doi: 10.1192/bjo.2025.10936 (PMC12835692; doi:10.1192/bjo.2025.10936)
Supplement: Tiley et al. supplementary material 1 — Tiley et al. supplementary material [file S2056472425109368sup001.pdf]

NHS
National Institute for
Health Research

UNIVERSITY of York
Centre for Reviews and Dissemination

Systematic review

A list of fields that can be edited in an update can be found here

1. * Review title.

Give the title of the review in English

The prevalence of autism spectrum traits and autism spectrum disorders in children and adolescents with obsessive compulsive disorder

2. Original language title.

For reviews in languages other than English, give the title in the original language. This will be displayed with the English language title.

3. * Anticipated or actual start date.

Give the date the systematic review started or is expected to start.

02/09/2020

4. * Anticipated completion date.

Give the date by which the review is expected to be completed.

14/05/2021

5. * Stage of review at time of this submission.

This field uses answers to initial screening questions. It cannot be edited until after registration.

Tick the boxes to show which review tasks have been started and which have been completed.

Update this field each time any amendments are made to a published record.

The review has not yet started: No

Review stage	Started	Completed
Preliminary searches	Yes	No
Piloting of the study selection process	Yes	No
Formal screening of search results against eligibility criteria	Yes	No
Data extraction	No	No
Risk of bias (quality) assessment	No	No
Data analysis	No	No

Provide any other relevant information about the stage of the review here.

6. * Named contact.

The named contact is the guarantor for the accuracy of the information in the register record. This may be any member of the review team.

Claire Tiley

Email salutation (e.g. "Dr Smith" or "Joanne") for correspondence:

Dr Tiley

7. * Named contact email.

Give the electronic email address of the named contact.

claire.tiley@slam.nhs.uk

8. Named contact address

Give the full institutional/organisational postal address for the named contact.

Croydon CAMHS, Christopher Wren House, 115 High Street, Croydon, Surrey, CR0 1QG

9. Named contact phone number.

Give the telephone number for the named contact, including international dialling code.

07867677147

10. * Organisational affiliation of the review.

Full title of the organisational affiliations for this review and website address if available. This field may be completed as 'None' if the review is not affiliated to any organisation.

South London and Maudsley NHS Foundation Trust

Organisation web address:

11. * Review team members and their organisational affiliations.

Give the personal details and the organisational affiliations of each member of the review team. Affiliation

refers to groups or organisations to which review team members belong. **NOTE: email and country now MUST be entered for each person, unless you are amending a published record.**

Dr Claire Tiley. South London and Maudsley NHS Foundation Trust
Dr Marinos Kyriakopoulos. South London and Maudsley NHS Foundation Trust

12. * Funding sources/sponsors.

Details of the individuals, organizations, groups, companies or other legal entities who have funded or sponsored the review.

None.

Grant number(s)

State the funder, grant or award number and the date of award

13. * Conflicts of interest.

List actual or perceived conflicts of interest (financial or academic).

None

14. Collaborators.

Give the name and affiliation of any individuals or organisations who are working on the review but who are not listed as review team members. **NOTE: email and country must be completed for each person, unless you are amending a published record.**

15. * Review question.

State the review question(s) clearly and precisely. It may be appropriate to break very broad questions down into a series of related more specific questions. Questions may be framed or refined using PI(E)COS or similar where relevant.

The aims of this systematic review are 1) To review studies that report on the prevalence of ASD traits and ASD diagnosis in children and adolescents with a diagnosis of OCD.

2) To review whether the severity of OCD symptoms is related to the severity of ASD traits in children and adolescents diagnosed with OCD.

3)To review whether the severity of co-morbid ASD traits in children and adolescents diagnosed with OCD impact on the child or adolescent's global functioning.

16. * Searches.

State the sources that will be searched (e.g. Medline). Give the search dates, and any restrictions (e.g. language or publication date). Do NOT enter the full search strategy (it may be provided as a link or attachment below.)

Three sources will be used which include MEDLINE, Embase and PsycINFO. The language will be restricted to English and the publication type to journal articles. There will be no restrictions on publication date.

17. URL to search strategy.

Upload a file with your search strategy, or an example of a search strategy for a specific database, (including the keywords) in pdf or word format. In doing so you are consenting to the file being made publicly accessible. Or provide a URL or link to the strategy. Do NOT provide links to your search **results**.

https://www.crd.york.ac.uk/PROSPEROFILES/218846_STRATEGY_20201120.pdf

Alternatively, upload your search strategy to CRD in pdf format. Please note that by doing so you are consenting to the file being made publicly accessible.

Yes I give permission for this file to be made publicly available

18. * Condition or domain being studied.

Give a short description of the disease, condition or healthcare domain being studied in your systematic review.

I will be looking at the prevalence of autism spectrum traits and autism spectrum disorder in children and adolescents with obsessive compulsive disorder.

Obsessive compulsive disorder is a disorder in which an individual experiences obsessions (repeated thoughts, impulses or images) and/or compulsions (the need to complete rituals or mental acts) which causes the individual functional impairment. Compulsions are performed to prevent anxiety or distress or a perceived dreaded event or situation. The symptoms are recognised as originating in the individuals own mind.

Autism Spectrum Disorder is a neurodevelopmental disorder characterised by three main domains impaired communication, impaired reciprocal social interaction and restricted and repetitive interests or patterns of behaviour.

19. * Participants/population.

Specify the participants or populations being studied in the review. The preferred format includes details of both inclusion and exclusion criteria.

Participants have to be age 18 and under and have a diagnosis of obsessive compulsive disorder. If the sample included adults where data about the adolescent sample can not be extracted this will be excluded.

[1 change] 20. * Intervention(s), exposure(s).

Give full and clear descriptions or definitions of the interventions or the exposures to be reviewed. The preferred format includes details of both inclusion and exclusion criteria.

For this review the participants will have to be up to the age of 18 and have a diagnosis of obsessive compulsive disorder (either International Classification of Disorders—ICD or the Diagnostic and Statistical Manual—DSM). Papers looking at disorders related to obsessive compulsive disorders including body dysmorphic disorder, compulsive skin picking, trichotillomania and hoarding disorder will not be included. Only papers that evaluate Autism Spectrum Disorder or Autistic Traits in young people with a diagnosis of Obsessive Compulsive Disorder will be included in this review.

21. * Comparator(s)/control.

Where relevant, give details of the alternatives against which the intervention/exposure will be compared (e.g. another intervention or a non-exposed control group). The preferred format includes details of both inclusion and exclusion criteria.

Where possible the OCD group will be compared to a control group or normative data.

22. * Types of study to be included.

Give details of the study designs (e.g. RCT) that are eligible for inclusion in the review. The preferred format includes both inclusion and exclusion criteria. If there are no restrictions on the types of study, this should be stated.

Any study where the relevant data can be extracted eg, epidemiological, case-control, cohort and RCTs

23. Context.

Give summary details of the setting or other relevant characteristics, which help define the inclusion or exclusion criteria.

The child or adolescent has to have received a diagnosis of OCD in any setting .

24. * Main outcome(s). [3 changes]

Give the pre-specified main (most important) outcomes of the review, including details of how the outcome is defined and measured and when these measurement are made, if these are part of the review inclusion criteria.

The first outcome will be the prevalence of ASD traits and ASD diagnosis in children and adolescents with a diagnosis of OCD. This will be measured by analysing ASD questionnaire score means and also ascertaining which meet the clinical cut off for ASD or scores which reflect the presence of ASD traits within a population of children and adolescents who have received a diagnosis of OCD. This will also be measured by reviewing the percentage of an OCD group who have also met the diagnostic criteria for ASD.

A second outcome will be the relationship between OCD severity and ASD trait severity in children and adolescents diagnosed with OCD. This will be done by correlating the questionnaire scores for ASD and OCD. We will also compare questionnaire scores between a co-morbid ASD and OCD group and and OCD only group.

A third outcome will be the relationship between ASD questionnaire scores and global functioning scores in a population of C&As with OCD. We will also compare questionnaire scores between a co-morbid ASD and OCD group and and OCD only group.

Measures of effect

Please specify the effect measure(s) for you main outcome(s) e.g. relative risks, odds ratios, risk difference, and/or 'number needed to treat.

Outcome 1 - measures of effect will include prevalence rates reported as a percentage or as mean ASD questionnaire aggregate scores. Where possible we will analyse the standardised mean difference (SMD), independent t-tests, relative risk or odds ratio where the prevalence of ASD traits or diagnosis in the OCD group is compared to a control group or normative data.

Outcome 2 - correlation between ASD questionnaire aggregate scores and OCD questionnaire aggregate scores using Pearson Correlation and/or regression analysis.

Outcome 3 - correlation between ASD questionnaire score and global functioning questionnaire scores using Pearson's correlation and/ or regression analysis.

For all 3 outcomes the measure of effect may be comparing a comorbid ASD and OCD group with an OCD only group which may be done by independent group t-test analysis, Standardised mean difference or ANOVA analysis using measure effects such as Cohen's d

25. * Additional outcome(s).

List the pre-specified additional outcomes of the review, with a similar level of detail to that required for main outcomes. Where there are no additional outcomes please state 'None' or 'Not applicable' as appropriate to the review

None.

Measures of effect

Please specify the effect measure(s) for you additional outcome(s) e.g. relative risks, odds ratios, risk difference, and/or 'number needed to treat.

Not applicable.

26. * Data extraction (selection and coding). [2 changes]

Describe how studies will be selected for inclusion. State what data will be extracted or obtained. State how this will be done and recorded.

The lead author will examine the abstracts of all publications identified by the search strategy using broad inclusion/exclusion criteria. The citations identified will either be excluded or evaluated further by obtaining the full text. The full text will be reviewed by the review authors to identify which studies meet both the inclusion and exclusion criteria. Conflict of opinion between authors regarding the eligibility of a study will be discussed with an independent reviewer.

In terms of inclusion criteria for this review, the participants will have to be up to the age of 18 and have a diagnosis of obsessive compulsive disorder (either International Classification of Disorders—ICD or the Diagnostic and Statistical Manual—DSM). Papers looking at disorders related to obsessive compulsive disorders including body dysmorphic disorder, compulsive skin picking, trichotillomania and hoarding disorder will not be included. Only papers that evaluate ASD or Autistic Traits in young people with a diagnosis of OCD will be included in this review. Papers will also be excluded if the sample includes adults where data about the adolescent sample cannot be extracted. Papers that are not written in English, poster abstracts and dissertations will be excluded.

Prevalence percentage figures will be extracted from each study either based on meeting the clinical cut off on ASD questionnaires or if scores indicate ASD traits. Reports on prevalence of ASD diagnosis within a pre-specified OCD sample will be included. Aggregate mean autism questionnaire scores will be extracted. In order to compare the relationship between OCD and ASD severity, these mean aggregate questionnaire scores will be correlated, this may include the SRS, SCQ, AQ, CY-BOCS, RCADS and TOCS. To compare the relationship between ASD severity in patients with OCD and global functioning we will analyse mean aggregate questionnaire scores such as the SRS, SCQ, AQ, CGI, COIS-P and HONOSCA.

27. * Risk of bias (quality) assessment.

State which characteristics of the studies will be assessed and/or any formal risk of bias/quality assessment tools that will be used.

The quality of each study will be assessed according to a standardised and validated set of criteria based on the protocols of the STROBE reporting guidelines.

28. * Strategy for data synthesis.

[1 change]

Describe the methods you plan to use to synthesise data. This **must not be generic text** but should be **specific to your review** and describe how the proposed approach will be applied to your data. If meta-analysis is planned, describe the models to be used, methods to explore statistical heterogeneity, and software package to be used.

We will tabulate the characteristics of each study including the sample size, age, gender and questionnaires included. The prevalence of ASD diagnosis in each OCD sample will be reported as a percentage and ASD traits as mean aggregate questionnaire scores. The table will include details on any comparison group data or normative data used. From these comparisons, the SMD, independent t-test, RR or OR will be reported where possible. The table will report on Pearson correlation assessing the correlation between (a) OCD severity and ASD trait severity and (b) ASD severity in the OCD sample and functional impairment.

From each study we will extract data on the prevalence of ASD traits/ diagnosis as a percentage or mean aggregate questionnaire score which should be analysed from a minimum of 8 studies. For the Pearson's correlations, a minimum of three studies should be included for each of the relationships assessed (a) and (b).

The data will only be analysed using meta-analysis if participants, methodology and outcome measures are sufficiently similar. If meta-analysis of any aspect of data is possible we will conduct it using the Review Manager software (random effects model). Mean aggregate scores from each study will be used. Prevalence rates, SMD, t-tests, RR, OR and Pearson's correlations across the studies will be analysed using a random effects model.

If the data isn't sufficiently similar, systematic narrative synthesis will be used. We will review prevalence rates, aggregate ASD mean questionnaire scores and where possible detail SMDs, t-tests, RR, OR and Pearson’s correlations - deriving the estimates of effect from each. We will investigate the similarities and differences between the findings of each study.

We will also examine the effects of clinical and methodological heterogeneity across all eligible studies using the tabulated information whilst assessing for bias.

29. * Analysis of subgroups or subsets.

State any planned investigation of ‘subgroups’. Be clear and specific about which type of study or participant will be included in each group or covariate investigated. State the planned analytic approach.

Not applicable

30. * Type and method of review.

Select the type of review, review method and health area from the lists below.

Type of review

Cost effectiveness
No

Diagnostic
Yes

Epidemiologic
Yes

Individual patient data (IPD) meta-analysis
No

Intervention
No

Living systematic review
No

Meta-analysis
Yes

Methodology
No

Narrative synthesis
Yes

Network meta-analysis
No

Pre-clinical
No

Prevention
No

Prognostic

No

Prospective meta-analysis (PMA)
No

Review of reviews
No

Service delivery
No

Synthesis of qualitative studies
No

Systematic review
Yes

Other
No

Health area of the review

Alcohol/substance misuse/abuse
No

Blood and immune system
No

Cancer
No

Cardiovascular
No

Care of the elderly
No

Child health
Yes

Complementary therapies
No

COVID-19
No

Crime and justice
No

Dental
No

Digestive system
No

Ear, nose and throat
No

Education
No

Endocrine and metabolic disorders
No

Eye disorders
No

General interest
No

Genetics
No

Health inequalities/health equity
No

Infections and infestations
No

International development
No

Mental health and behavioural conditions
Yes

Musculoskeletal
No

Neurological
No

Nursing
No

Obstetrics and gynaecology
No

Oral health
No

Palliative care
No

Perioperative care
No

Physiotherapy
No

Pregnancy and childbirth
No

Public health (including social determinants of health)
No

Rehabilitation
No

Respiratory disorders
No

Service delivery
No

Skin disorders
No

Social care

No

Surgery
No

Tropical Medicine
No

Urological
No

Wounds, injuries and accidents
No

Violence and abuse
No

31. Language.

Select each language individually to add it to the list below, use the bin icon to remove any added in error.
English

There is an English language summary.

32. * Country.

Select the country in which the review is being carried out. For multi-national collaborations select all the countries involved.

England

33. Other registration details.

Name any other organisation where the systematic review title or protocol is registered (e.g. Campbell, or The Joanna Briggs Institute) together with any unique identification number assigned by them. If extracted data will be stored and made available through a repository such as the Systematic Review Data Repository (SRDR), details and a link should be included here. If none, leave blank.

34. Reference and/or URL for published protocol.

If the protocol for this review is published provide details (authors, title and journal details, preferably in Vancouver format)

Add web link to the published protocol.

Or, upload your published protocol here in pdf format. Note that the upload will be publicly accessible.

No I do not make this file publicly available until the review is complete

Please note that the information required in the PROSPERO registration form must be completed in full even if access to a protocol is given.

35. Dissemination plans.

Do you intend to publish the review on completion?

Yes

Give brief details of plans for communicating review findings.?

Through scientific conferences and publication in a peer review journal.

36. Keywords.

Give words or phrases that best describe the review. Separate keywords with a semicolon or new line. Keywords help PROSPERO users find your review (keywords do not appear in the public record but are included in searches). Be as specific and precise as possible. Avoid acronyms and abbreviations unless these are in wide use.

obsessive compulsive disorder; ocd; autis*; autism trait;
Asperger*; ASD; child*; adoles*; p?diatric*; youth; juvenile; teen*; young people; young person.

[1 change]
37. Details of any existing review of the same topic by the same authors.

If you are registering an update of an existing review give details of the earlier versions and include a full bibliographic reference, if available.

38. * Current review status.

Update review status when the review is completed and when it is published.New registrations must be ongoing so this field is not editable for initial submission.
Please provide anticipated publication date

Review_Ongoing

[1 change]
39. Any additional information.

Provide any other information relevant to the registration of this review.

We believe that this systematic review is important as children and adolescents with OCD have been identified as having higher rates of co-morbid psychiatric diagnosis in comparison to the general population of children and adolescents. Children and adolescents with co-morbid OCD and ASD may require tailored treatment, specifically cognitive behavioural therapy adapted to ASD. Therefore, early identification of ASD in these individuals may be beneficial.

40. Details of final report/publication(s) or preprints if available.

Leave empty until publication details are available OR you have a link to a preprint (NOTE: this field is not editable for initial submission). List authors, title and journal details preferably in Vancouver format.

Give the link to the published review or preprint.
