# Supplementary material for: The prevalence of autism spectrum traits and autism spectrum disorders in children and adolescents with obsessive compulsive disorder: systematic review and meta-analysis
Source: BJPsych Open. 2026 Jan 16;12(1):e39. doi: 10.1192/bjo.2025.10936 (PMC12835692; doi:10.1192/bjo.2025.10936)
Supplement: Tiley et al. supplementary material 2 — Tiley et al. supplementary material [file S2056472425109368sup002.docx]

| **Questionnaire (include reference)**  **Supplementary Table 2 – Questionnaire Characteristics** | **Rater** | **Age/ Time period** | **Subscales** | **Total Items** | **Cut-off score** | **Scoring** |
| --- | --- | --- | --- | --- | --- | --- |
| Social Communication Questionnaire (SCQ) (1) | Caregiver | Current  Lifetime | Reciprocal social interaction- 20 items  Language and communication – 11 items  Stereotyped patterns of behaviour – 8 items | 40 | 15 | Dichotomous - yes/no answers |
| Social Responsiveness Scale (SRS)(2) | Caregiver | Age 4-18 | Social Awareness – 8 items  Social Cognition – 12 items  Social Communication -22 items  Social motivation -11 item  Restricted interests and repetitive behaviour -12 items | 65 | 60-75 = mild-moderate ASD symptoms  >76= severe ASD symptoms | Scored on Likert Scale  Not true=1, sometimes true =2, often true=3, almost always true=4 |
| Social Responsiveness Scale – second edition (SRS-2) (3) | Caregiver | Age 2.5- adulthood | Social Awareness – 8 items  Social Cognition – 12 items  Social Communication -22 items  Social motivation -11 item  Restricted interests and repetitive behaviour -12 items | 65 | 60-75 = mild-moderate ASD symptoms  >76= severe ASD symptoms | Scored on a 4 point Likert Scale  Not true=1, sometimes true =2, often true=3, almost always true=4 |
| Autism Spectrum Screening Questionnaire (ASSQ) (4, 5) | Caregiver | Aged 7-16 | Social interaction (11 items)  Communication Problems (6 items)  Restricted and repetitive behaviour (5 items) | 27 items | Two levels of cut off -  >/=17  >/=13 | Rated on a three-point Likert scale:  “not true” (0), “somewhat true” (1), and “certainly true” (2). |
| Autism Quotient - (AQ) (6) | Child and adolescent version – caregiver  Adult – self administered | Child (4-11)  Adolescent (12-15)  Adult | Social Skills – 10 items  Attention switching – 10 items  Attention to detail – 10 items  Communication – 10 items  Imagination – 10 items | 50 items | Threshold score =26  Common cut-off 32 | Rated on a 4-point Likert scale “definitely agree.” “slightly agree,” “slightly disagree,” ”definitely disagree” |
| Chldren’s Social Behaviour Questionnaire (CSBQ)(7) | Care giver/ Parent | Aged 4-18 | Behaviour/ emotions not optimally tuned to the social situation  Reduced contact and social interest  Orientation problems in time, place or activity  Difficulties in understanding social information  Stereotyped behaviour  Fear and resistance to changes | 49 items | Normoscore of 6 | Raw score converted to normoscore ranging from 0 (very low) to 6 (very high) |
| The Children’s Yale Brown Obsessive Compulsive Scale (CY-BOCS)(8) | Clinician rated semi-structured interview | Aged 6-17 | Obsession subscale  Compulsion subscale  Each subscale examines:  -Frequency/time  -Interference  -Distress  -Resistance  -Control | 10 items | 0-7 – Subclinical  8-15 – Mild OCD Symptoms  16-23 – Moderate OCD Symptoms  24-31 - Severe OCD  Symptoms  32-40 – Extreme OCD Symptoms | Items are rated by the clinician on a five-point scale from  0 to 4. |
| Child Obsessive-Compulsive Impact Scale -parent version (COIS-P) (9) | Parent/ Caregiver | Aged 5-17 | 3 domains:  -Social  -School  -Family/Home | 20 items | N/A | Rated on a 4-point Likert Scale.  0=Not at all, 1=Just a little, 2=Pretty much, 3=Very much. |
| Children’s Global Assessment Scale (CGAS) (10) | Clinician rated | Children and adolescents | Ratings are based on the level of emotional and behavioural functioning over the past 3 months. | N/A | <70 indicates some impairment of functioning | Scored from 1-100 |

ASD-Autism Spectrum Disorder, OCD – Obsessive Compulsive Disorder

**References**

1. Rutter M, Bailey A, Lord C. The Social Communication Questionnaire. Los Angeles: Western Psychological Services. 2003.

2. Constantino JN. Social Responsiveness Scale. In: Volkmar FR, editor. Encyclopedia of Autism Spectrum Disorders. New York, NY: Springer New York; 2013. p. 2919-29.

3. Constantino JN, Gruner P. Social Responsiveness Scale, Second Edition (SRS-2). Torrance, CA: Western Psychological Services. 2012.

4. Ehlers S, Gillberg C. The epidemiology of Asperger syndrome. A total population study. J Child Psychol Psychiatry. 1993;34(8):1327-50.

5. Ehlers S, Gillberg C, Wing L. A screening questionnaire for Asperger syndrome and other high-functioning autism spectrum disorders in school age children. J Autism Dev Disord. 1999;29(2):129-41.

6. Baron-Cohen S, Wheelwright S, Skinner R, Martin J, Clubley E. The autism-spectrum quotient (AQ): evidence from Asperger syndrome/high-functioning autism, males and females, scientists and mathematicians. J Autism Dev Disord. 2001;31(1):5-17.

7. Hartman CA, Luteijn E, Serra M, Minderaa R. Refinement of the Children's Social Behavior Questionnaire (CSBQ): an instrument that describes the diverse problems seen in milder forms of PDD. J Autism Dev Disord. 2006;36(3):325-42.

8. Scahill L, Riddle MA, McSwiggin-Hardin M, Ort SI, King RA, Goodman WK, et al. Children's Yale-Brown Obsessive Compulsive Scale: reliability and validity. J Am Acad Child Adolesc Psychiatry. 1997;36(6):844-52.

9. Piacentini J, Peris TS, Bergman RL, Chang S, Jaffer M. Functional impairment in childhood OCD: development and psychometrics properties of the Child Obsessive-Compulsive Impact Scale-Revised (COIS-R). J Clin Child Adolesc Psychol. 2007;36(4):645-53.

10. Shaffer D, Gould MS, Brasic J, Ambrosini P, Fisher P, Bird H, et al. A children's global assessment scale (CGAS). Arch Gen Psychiatry. 1983;40(11):1228-31.
