# Supplementary material for: The prevalence of autism spectrum traits and autism spectrum disorders in children and adolescents with obsessive compulsive disorder: systematic review and meta-analysis
Source: BJPsych Open. 2026 Jan 16;12(1):e39. doi: 10.1192/bjo.2025.10936 (PMC12835692; doi:10.1192/bjo.2025.10936)
Supplement: Tiley et al. supplementary material 3 — Tiley et al. supplementary material [file S2056472425109368sup003.docx]

| **Paper** | **Country of study, Sample,**  **Supplementary Table 3 - Data Extraction Table**  **Male: Female Ratio, comparison group and study design** | **Mean age in years, mean age of onset or age range** | **Questionnaire measures** | **Results- prevalence of ASD traits/ ASD diagnosis in individuals with OCD** | **Results – correlation of OCD severity and ASD traits** | **Results – functional impairment in OCD related to severity of ASD traits** | **Comments** |
| --- | --- | --- | --- | --- | --- | --- | --- |
| Adam et al. (2019)(1) | Total OCD sample diagnosed according to ICD-10 criteria (n=181)  Male, n= 96 (53%), Female, n=85 (47%)  Total OCD sample aged 6-10 (n=46).  Total OCD sample aged 11-18 (n=135)  Case-control Study | Total OCD sample mean age:13.0, SD=3.0  Total OCD sample age range:6-18.0 | CY-BOCS | 5% (n=9) of the total sample diagnosed with OCD also had a diagnosis of PDD. | Not reported on | Not reported on | The main purpose of the study was to assess the psychometric properties of the German Version of the Padua Inventory-Washington State University Revision for measuring paediatric OCD |
| Arildskov et al. (2016)(2) | Denmark, Norway & Sweden  Total OCD sample diagnosed according to DSM-IV-TR criteria: (n= 257)  Male n=125 (48.6%)  Female n = 132 (51.4%) of total OCD sample.  Normative data from Ehlers and Gillberg (n=1401)  Cross-sectional but participants originally recruited as part of a multi-step intervention study including RCT. | OCD Sample Mean Age: 12.79 SD=2.75  OCD Sample Age of onset: 11.56 SD=3.01 | ASSQ  CY-BOCS | OCD group (not excluding comorbid dx): mean ASSQ score = 7.18 SD= 6.93  Normative data: mean ASSQ score = 0.70 SD=2.58  Mann Whitney U – as data not normally distributed -U = 33,096.00, z = −25.03, p < .001, t=2.62, sd=36.52 a large effect size  r = .61  ---------------------------------------  Pure OCD group (excluding comorbid dx)-n=160. Mean ASSQ score=5.47, SD= 5.19, Median=4.  Normative data: mean ASSQ score = 0.70 SD=2.58    Mann Whitney U – U = 26,438.00, z = − 19.91, p < .001, a large  effect size r = .50.  ---------------------------------------  OCD Group: ASSQ score >or= to 17-n =25 (9.7%), SD =9.7  Normative Group: ASSQ score>or= to 17 = 8 (0.6%), SD=0.6  Pearson’s Chi square test- compare differences in proportions - χ2(1, N = 1658) = 93.34,  p < .001, a small effect size rϕ = .24  ---------------------------------------  OCD Group ASSQ score>or= to 13 = 44, SD=17.1 (25.6% of male OCD sample compared to 9.1% of female OCD sample)  Normative Group: ASSQ score >or= to 13 =10 SD=0.7  Pearsons’s Chi square test-  χ2(1, N = 1658) = 185.53,  p < .001, a medium effect size rϕ = .34. | Three -step multiple regression analysis model.  Significant correlation between mean CY-BOCS scores and mean ASSQ F(10,242)=2300, p=0.014.  The motor/tic/OCD subscale was the only variable that significantly contributed. This subscale was positively correlated to OCD severity B=.514, 95% CI [.184,.844] t(242)=3.064 p=0.002.  The autistic style and social difficulties subscales did not contribute significantly to the model as these autism specific symptoms did not significantly correlate with the mean CY-BOCS score.  Autistic style (β = −.065, 95 % CI [−.417, .287],  t(242) = −.361, p = .72) not statistically significant and Social difficulties (β = .074,  95 % CI [−.182, .330], t(242) = .566, p = .57) no statistically significant. | Not reported on. | Limitations  CY-BOCS score and motor/tics/OCD subscale on ASSQ measure the same construct which may affect ability to differentiate between OCD and ASD.  Queries were raised by the authors as to whether ASSQ too sensitive a tool. |
| Boedhoe et al.(2020)(3) | Worldwide  Total OCD Sample n=479  Paediatric OCD Sample – n=140  Male = n=76 (54.29%) and Female – n=64 (45.71%)  Adolescent OCD Sample – n=339  Male – n=195 (54.32%) and Female – n=164 (45.68%)  Case-control Study | Paediatric OCD sample mean age =10.28, SD=2.2  Adolescent OCD sample mean age=14.91, SD=1.72. | - | 2.14% (n=3) of the paediatric population with a diagnosis of OCD, were also diagnosed with ASD.  2.95% (n=10) of the adolescent population with a diagnosis of OCD, were also diagnosed with ASD.  2.71% (n=13) of the combined population with a diagnosis of OCD, were also diagnosed with ASD. | Not reported on | Not reported on | Samples taken from the ENIGMA ASD working group and OCD working group  Lack of clarification over which diagnostic criteria was used to diagnose OCD and ASD |
| Chen et al. (2022)(4) | Taiiwan  Total child and adolescent (under 18) OCD Sample – n=4726 diagnosed according to ICD-9 CM. Male-n=2991 (63.3%), female n=1735 (36.7%), mean age=14.96, sd=2.10,  Longitudinal Follow Up Study  Country: Taiwan | Child and adolescent (under 18) mean age=14.96, sd=2.10, | - | 9.39 % (n=444) of the paediatric population with a diagnosis of OCD, were also diagnosed with ASD. | Not reported on | Not reported on | Study focussed on the rates of progression of schizophrenia in children, adolescents and adults with a diagnosis of OCD and the effects of co-morbidities.  Limitation:  Sample started from age 10, mean age 14.96 within child and adolescent sample. |
| Choi et al. (2021)(5) | Canada  Total OCD sample diagnosed according to DSM-IV - n=55. Male-n=35 (63.6%), Female-n=20 (36.4%)  Control Group (Typically Developing Children) – n=84. Male – n=48 (57.1%), Female – n=36 (42.9%).  Single Cohort Study | Total OCD Sample Mean Age: 12.94, SD=2.84 | SCQ | OCD Group: SCQ mean score =6.22, SD=5.62, n=55  Control Group: SCQ mean score=2.19, SD=2.29,n=84  Comparison= t=5,9 , sd=0.7 F=174,45 , df = 3.346 , post hoc ASD > ADHD,OCD > TD | Not reported on | Not reported on | Initial study was investigating canonical resting-state functional networks in three diagnostic groups (ASD, ADHD and OCD) and a TD sample. |
| Farrell et al. (2012)(6) | Australia  Total OCD sample diagnosed according to DSM-IV- n=43. Male -n=30 (70%), Female -n=13 (30%)  Case-Control Study | Total OCD Sample Mean Age: 11.1, SD=2.52  Total OCD age range 7-17 |  | 35% (n=15) of the sample diagnosed with OCD were also diagnosed with PDD | Not reported on | Not reported on | Study evaluated the effectiveness of a group CBT intervention for children and adolescents with OCD and co-morbidity. Specifically the impact of comorbidity on response and remission rates to treatment.  Limitations – small sample size. Pilot study – no comparison to control group. |
| Geller et al. (1996)(7) | USA  Total OCD sample diagnosed according to DSM-III-R criteria – n=30. Male – n=21 (70%), Female -n=9 (30%).  Cross-Sectional Study | Total OCD Sample Mean Age:12.6, SD=2.9 | CY-BOCS | 6.7% (n=2) of the sample diagnosed with OCD also received a diagnosis of PDD. | Not reported on | Not reported on | This study examined a range of co-morbidities in juveniles with OCD and compared these to other similar studies  Limitations: Small sample. OCD samples were referred, query whether this isrepresentative of non-referred OCD samples. |
| Griffiths et al. (2017)(8) | Australia  Total OCD sample diagnosed according to DSM-IV criteria - n=80  Male - n = 38 (45%)  Female - n =44 (55%)  Normative data of SRS-2 scores -standardised sample- n =2025 Aged 4-18 years.  Cross-sectional study | Total OCD Sample Mean Age: 12.30 SD=2.68 | SRS-2  CY-BOCS  COIS-P | ASD prevalence in OCD group = 5%  n=4  ---------------------------------------  57.5% of OCD sample had elevated ASD traits above normal range (t>60) – n= 48.  32.5% of OCD sample were above the cut off for moderate – severe ASD symptoms – n=26.  SRS total mean score in the OCD total sample – m=60.41 SD=9.00,n=80  SRS total mean scores in normative standardised group m=30.90 SD=25.30,n=2025  Comparison=  t=10.40, sd=2.84 | Pearson’s correlation used  Correlation between CY-BOCS total severity score and SRS total T score was not statistically significant (r= -.13, p=.16). | Pearson’s correlation used  COIS-P total impact score was positively correlated with the SRS total t-score (r=.23, p=.02)  SRS total t-score was a significant predictor of OCD-related functional impairment as measured by COIS-P  (R2 chg=0.05, Fchg (1, 77)=4.03, p=.05, B=0.63, SE=0.32). | The study compared two age groups 7-12 and 13-17 years.  The study also measured family accommodation scores.  Limitation – predominantly mid-high social-economic status and high levels of education.  Structured psychometrically validated interviews to screen for ASD were not used.  Queries whether SRS is too sensitive to differentiate OCD and ASD. |
| Griffiths et al. (2017)(9) | Australia  Total OCD sample diagnosed according to DSM IV criteria – n=117.  OCD + ASD group - n =25  Male -n=22 (88%)  Female -n =3 (12%)  OCD only group - n=25  Male - n =22 (88%)  Female - n = 3 (12%)  Case-control study | Age range of total sample = 7-17 years.  OCD + ASD group n=25, mean age =12.12 SD=2.30.  OCD only group n= 25, mean age =11.92 SD=2.56 | SRS-2  CY-BOCS  COIS-P | Overlap of sample with Griffiths et al paper -ASD Traits Among Youth with Obsessive–Compulsive Disorder -2017 | No significant differences between total CY-BOC scores or subscales between the OCD only group vs. OCD +ASD group.  CY-BOC total mean score in OCD only sample m=23.08, SD=6.8 n=25  CY-BOC total mean score in OCD + ASD group, m=23.50, SD=6.42, n=25  Total CY-BOC score group comparison  t=0.22, p=0.83 | Independent group t-tests showed significant differences between the OCD only group vs. OCD+ASD group.  COIS-P total mean score in OCD only sample, m=31.87, SD=14.97, n=25  COIS-P total mean score in OCD + ASD sample, m=51.82, SD=23.26, n=25  Total COIS-P group comparison =t(35)=3.41, p=0.002, Cohen’s d =1.02  School functioning t(42)=2.03, p=0.05, Cohen’s d=0.61.  Social functioning t(36)=2.58, p=0.01, Cohen’s d =0.77.  Home and family activities t(43)=2.64,p=0.01, Cohen’s d = 0.79. | Co-morbidities, family accommodation and parental psychopathology were also compared.  Treatment responses to CBT between groups were also compared – no significant differences were found post treatment.  Limitations - study did not use structured psychometrically validated interviews for a diagnosis of ASD although semi-structured clinical interview questions were used and ASD diagnosis was confirmed by external paediatric specialist.  88% of the sample were male.  There was a reliance on parental reporting of symptoms.  OCD + ASD group had significantly more comorbid symptoms than OCD alone group which may confound the results. |
| Hanna (1994)(10) | USA  Total OCD sample diagnosed according to DSM-III-R – n=31  Male -n=19 (61%), Female -n=12 (39%)  Cross-Sectional Study | Total OCD sample mean age=13.5, SD=2.8.  Total OCD sample age range 7.7-18.0 | CY-BOCS | 3.2% (n=1) of the sample dignosed with OCD also received a co-morbid diagnosis of PDD.  100% of those diagnosed with PDD were male | Not reported on | Not reported on | The study looked at co-morbidity of children and adolescents with a diagnosis of OCD, including OCD severity and demographic information.  Limitations: small sample size |
| Hojgaard et al. (2023)(11) | Denmark, Norway and Sweden  Total OCD sample diagnosed according to DSM-IV criteria n=257  OCD only group- n=232  Male – n=107 (46%), Female-n=125(54%)  OCD + autism trait group – n=25  Male-n=11 (44%), female-n=14 (56%)  Case-control study | Total OCD sample mean age =12.51, SD=2.77  Total OCD sample age range 7-17 | ASSQ  CY-BOCS | ASSQ total mean score=7.18, SD=4.79, n=257  For purposes of this meta-analysis –OCD data compared against ASSQ normative data.  Normative data: mean ASSQ score = 0.70 SD=2.58, n=1401  SMD = 2.14 (95%CI= 1.99-2.29)  Participants who had ASSQ >/=17 defined as having autistic traits – n=25 (9.73%)  In the higher ASD trait group 72% of the sample were male compared to the OCD only group where 46.1% were male. | OCD + autistic trait group CY-BOCS total mean score =26.56, sd=4.58, n=25  OCD only group CY-BOCS total mean score=24.50, sd=5.13, n=232  Comparison: odds ratio 1.09, 95% CI 1.00-1.19, p value=0.035 | OCD + autistic trait group clinical global impression scale (CGI-S) mean score =3.64,SD=0.81,n=25  OCD only group clinical global impression scale (CGI-S) mean score=3.42, SD=0.83, n=232  Comparison -odds ratio=1.48, 95% CI 0.85-2.63, p value=0.152 | CBT found to be equally effective for those with or without autisitic traits  Limitation – homogeneity of the Scandinavian sample which could limit generalisability. |
|  |  |  |  |  |  |  |  |
| Ivarsson et al.(2008)(12) | Sweden  Primary OCD sample diagnosed according to DSM-IV n=109  Male - n= 49 (45%)  Female - n = 60 (55%)  Comparison - general population data from Ehlers et al 1997 N=1401  Pure OCD group compared to OCD+ASD, OCD+ADHD, OCD+ tic syndrome, OCD+LD  Case-control study | Adolescent sample- n= 66  Children’s sample – n=49 | ASSQ  ASQ  CY-BOCS | ASSQ total score in this OCD sample – mean =7.86 SD=6.19, n=109  Normative data: mean ASSQ score = 0.70 SD=2.58, n=1401  Comparison – t=13.33  ---------------------------------------  ASSQ score when removing OCD and TS like items in the ASSQ -mean=4.6949 SD =4.80498  ---------------------------------------  8.26% of OCD group scored significantly higher than those with other diagnosis  ---------------------------------------  ASSQ score in ASD + OCD group – m=17.91 SD =8.38  ASSQ score in OCD only group – m=5.4, SD=4.05,  ---------------------------------------  Gender Differences- Male sample (n = 49) (M = 5.6, S.D. = 5.37) scored higher than female sample (n = 60) (M = 3.9, S.D. = 4.19) a difference approaching statistical significance (t(107) = 1.83, p = .071). | Spearman’s correlation used  The measures of OCD severity were not significantly correlated with the ASSQ-R: CYBOCS total score (r= .02, n.s.). | Not reported on | Limitations – The use of ASSQ and clinical interviews may mean some symptoms or traits are an artefact of assessment.  The measure is dependent on parental observations and interpretation. |
| Jaspers-Fayers et al. (2017)(13) | Canada  Total OCD sample diagnosed according to DSM-IV- n=106  Male-n=59 (56%), Female=47 (44%)  Cross-Sectional Study | Mean age of Total OCD sample=13.4, SD=3.1  Total OCD sample age range=6.0-18.0 | CY-BOCS | 6.6% (n=7) of total OCD sample were diagnosed with PDD | Not reported on | Not reported on | Study looked at the prevalence of PANDAS and PANS in paediatric OCD.  The published paper reported on total OCD sample n=136 aged 6-19 but authors provided data on those aged 6-18 with OCD including the number diagnosed with PDD. |
| Jassi et al.  (2021)(14) | United Kingdom  Total OCD sample diagnosed according to ICD-11- n= 619. Male=  OCD only sample – n=447.  OCD + ASD sample – n=172  ASD diagnosed according to DSM-IV criteria  Part of a case-control study | Total OCD Sample Mean Age=14.6, SD=2.2  Total OCD Sample Age range =6.0-18.0 | CY-BOCS  C-GAS | Prevalence of ASD diagnosis =27.8%  Those in the OCD +ASD group were more likely to be male than those in the OCD only group (OR = 0.52, 95% CI 0.36, 0.75) | OCD only group – mean CYBOCS score =27.6, SD=4.9, n=447  OCD + ASD group – mean CYBOCS score=29.09, SD=4.9, n=172  CYBOCS comparison between OCD vs OCD + ASD group – t=-1.08 SD=-15.42, p=0.282 | Sample overlap with Martin et al.’s study.  Information from Martin et al’s study used due to larger sample size. | This study assessed treatment outcomes in an OCD only vs OCD +ASD group following a course of Cognitive Behaviour Therapy*.*  Limitation – lack of diagnostic assessment for existing co-morbidities. |
| Lewin et al. (2011)(15) | United States of America (USA)  Total OCD sample, diagnosed according to DSM-IV– n=70.  OCD only group –n=35  OCD + ASD group – n=35  ASD diagnosed according to DSM-IV.  Case control-study | Mean age of total OCD sample = 9.9, SD=1.8  Age range of total OCD sample =7.0-13.0. | CY-BOCS | Not reported on | OCD only group – mean CYBOCS score =25.9, SD=5.7, n=35  OCD + ASD group – mean CYBOCS score=26.5, SD=8.4, n=35  CYBOCS comparison between OCD vs OCD + ASD group – t=0.34 SD=1.7 | Not reported on. | ADHD, separation anxiety and social phobia were more common in the group with both OCD+ASD.  Limitations –no ASD only group was available.  No data on functional impairment between groups was available. |
| Mahjoob et al. (2024)(16) | Canada  Total OCD sample diagnosed according to DSM-IV – n=38  Male – n= 21(55%), Female-n=17 (45%)  Cross-Sectional Study | Mean Age of total OCD sample =12.87, SD=2.72 | SCQ TOCS | 13% (n=5) of OCD sample scored >/= 11 on SCQ which was listed as cut-off score for elevated ASD traits  ---------------------------------------  1% (n=2) of TD sample scored >/= 11 on SCQ which was listed as cut-off score for elevated ASD traits  This paper also compared mean total SCQ scores between OCD sample and TD sample but potential overlap of sample with Choi 2020 and Baribeau 2019 | Not reported on | Not reported on | Main purpose of the study was to assess the predictors of health-related quality of life particularly focusing on patients with ADHD, Autism, OCD, subADHD, subOCD or who were typically developing.  Limitations: cross-sectional nature of data. |
| Martin et. al. (2020)(17) | United Kingdom  Total Sample (OCD only + ASD only + comorbid OCD+ASD)- n=7922  OCD + ASD sample -diagnosis confirmed according to ICD-10 criteria- n=335  Male - n=214 (64%)  Female - n =121 (36%)  OCD only sample (diagnosis confirmed according to ICD-10 criteria)- n=1010  Male - n=488 (48%)  Female - n=522(52%)  Comparison-  ASD only (diagnosis confirmed according to ICD-10 criteria)- n=6577  Retrospective cohort study | Age at OCD dx in the OCD + ASD group: 13.47 SD=2.81  Age at OCD dx in the OCD only group = 14.00 SD=2.59  Age at ASD dx in the OCD +ASD group:13.28 (3.01)  Age at ASD dx in the ASD only group =  10.76 SD =3.72 | RCADS  CGAS | 24.9% of patients with a diagnosis of OCD, were also diagnosed with ASD. | Duplication of sample with Jassi et al. Data from Jassi et al. used.  Mean ( +/- SD) RCADS OCD subscale score in  OCD +ASD group= 7.48 (5.29)  Mean ( +/- SD) RCADS OCD subscale score in  OCD group = 9.30 (5.26)  Comparison RCADS OCD subscale t values of OCD + ASD vs. OCD – t= -2.05, p<0.05 | Mean ( +/- SD) CGAS score in OCD +ASD group=44.30 (3.29)  Mean ( +/- SD) CGAS score in OCD group=  49.06 (15.57)  Comparison CGAS t values of OCD + ASD vs. OCD - t= -5.35  Improvement in functioning over time reduced in OCD +ASD vs OCD | The study explored the prevalence of Intellectual Disability, medication use and the use of CBT in OCD only group vs. a comorbid ASD + OCD group. |
| Memis et al.  (2019)(18) | Turkey  OCD adolescent sample diagnosed according to DSM -IV criteria- n=29  Male - n =15 (51.7%)  Female -n=14 (48.3%)  Comparison -Adult OCD sample diagnosed according to DSM -IV criteria- n= 45  Case-control study | Total OCD Sample Mean Age= 14.5, SD=2.1 | AQ  CY-BOCS Y-BOCS | Mean AQ total score in adolescent OCD group=19.1, SD=5.9  ---------------------------------------  Mean social skills subscale score in adolescent OCD group = 3.6 SD=1.8  ---------------------------------------  Mean attention shifting subscale score in adolescent OCD group = 4.4 SD=1.9  ---------------------------------------  Mean attention to detail subscale score in adolescent OCD group = 4.8 SD=2.3  ---------------------------------------  Mean communication subscale score in adolescent OCD group = 2.6 SD=2.1  ---------------------------------------  Mean imagination subscale score in adolescent OCD group=3.5, SD=1.9 | Spearman correlation used  Correlation between AQ total and CYBOCS total score r=0.17- p>0.05.- not statistically significant  Correlation between Communication subscale score on AQ and total obsession score r=0.48 p=0.007 – statistically significant.  Correlation between Communication subscale score on AQ and total compulsion score r=0.40 p=0.02 – statistically significant.  Correlation between Communication subscale score on AQ and CY-BOCS total score r=0.15 p>0.05 not statistically significant. | Not reported on | The main purpose of the study is to compare adolescent onset OCD with adult onset OCD in terms of subthreshold autistic traits.  Limitations – the small sample size may prevent generalisation of the findings.  A high proportion of both samples were taking antidepressant medication at the time of assessment. |
| Onat et al. (2019)(19) | Turkey  Total OCD sample diagnosed according to DSM-5 criteria n=33  Male OCD sample –n= 10 (30.3%)  Female OCD samoke – n=23 (69.7%)  Total Healthy Control Group n=35  Male Helathy Control Group – n=15 (42.9%)  Female Healthy Control Group – n=20 (57.1%)  Case-Control Study | Total OCD Sample median age =15 (IQR 3.63)  Total Healthy Control Sample Mesian Age =16 (IQR 3) | AQ-Adolescent  CY-BOCS  CGI | Median Total AQ Adolescent total score in OCD sample =20.0 IQR=7.0  Median Total AQ Adolescent total score in healthy control group =15.0 (IQR 14.0) | Significant positive correlation between the  CY-BOCS total score and the AQ-Adolescent total score (p=0.28) p<0.05 in the OCD group using Spearman’s correlation.  Table also appears that r=.423 p<0.05 | OCD sample CGI-S median score =5.0 IQR=1.0.  AQ total score median 20.0 IQR 7.0 | This study also compares AQ-adolescent scores between an OCD group and trichotillomania group, correlations between CY_BOCS, AQ and RBS-R scores in an OCD group and correlations between CGI-S, AQ scores and RBS-R scores in a trichotillomania sample. |
| Ozyurt et al. (2018)(20) | Turkey  OCD sample diagnosed according to DSM-V criteria- n=38  Male - n=23 (60.5%)  Female - n =15 (39.5%)  Age and gender matched control group - n= 39  Case-control study | Total OCD Sample Mean Age = 12.37, SD=2.77 | SCQ  CY-BOCS | Mean total SCQ score in OCD group = 6.92 SD = 3.89, n=38  Mean total SCQ score in control group=4.41SD=3.63, n=39  Compared by Mann Whitney U  Z= -2.872  P=0.004  Cohen’s d=0.667  Effect size=0.316  Gender differences  OCD group was divided as female and male, and compared by using Mann Whitney U test; Male sample obtained statistically significant higher scores in terms of SCQ total score , social interaction score, and stereotypical behaviors subscales (p=0.006, p=0.012, p=0.007, respectively) compared to the female sample. | Spearman’s correlation used  Correlation of total SCQ score with CY-BOCS score in OCD group r=0.08, p=0.632 – no statistical significance  Correlation of total SCQ score with CY-BOCS score in control group r= -0.197, p= 0.222 – no statistical significance. | Not reported on | Study assessed ASD symptoms and OCD in mothers of both the OCD and control groups.  The OCD group was divided as 8–12 and 13–18 in terms of age and compared.  Limitations –  Difficulties in differentiation ASD and OCD symptoms due to similarities in repetitive behaviour. |
| Peris et al. (2017)(21) | USA  OCD Sample diagnosed according to DSM-IV-T, n=322  Male – n=170 (53%)  Female – n=148 (47%)  Cross-Sectional Study | Total OCD Sample Mean Age =12.28, SD=2.75  OCD sample aged 7-9 – n=58  OCD sample aged 10-13 – n=148  OCD sample aged 14-17 – n=116 | CY-BOCS | 3% of total OCD sample met criteria for ASD  4% of male OCD sample met criteria for ASD  1% of female OCD sample met criteria for ASD  6% of OCD sample aged 7-9 met criteria for ASD  1% of OCD sample aged 10-13 met criteria for ASD  3% of OCD sample aged 14-17 met criteria for ASD | Not reported on | Not reported on | Study reported on prevalence of co-morbiditities in an OCD sample including anxiety disroders, depressive disorders and externalising disorders.  Study also predicted patterns of co-morbidity – predicting comorbidity type and explore most common classes of co-morbidity.  Limitations – lack of data of age of onset of OCD. Clinicians rotating frequently which mayaffect vartiability of the assessment process. |
| Perez-Vigil et al. (2021)(22) | Spain  Total OCD sample according to DSM-V criteria, n=19  Male- n=19 (100%)  Case-Control Study | Mean age of OCD sample=13.8  Age range of OCD sample =11.0-17.0 | CY-BOCS  ASSQ | Mean total ASSQ score in OCD group = 9.3 SD = , n=19  Mean total SCQ score in control group=2.0 SD=, n=20 | Not reported on | Not reported on | ASSQ, CY-BOCS and YGTTS compared between OCD, TS, ASD and control group  The study also compared theory of mind between the groups  Limitation – small sample sizes and all male samples |
| Salemink et al. (2023)(23) | Netherlands  Total OCD sample diagnosed according to DSM-IV, n=36. Male – n=23 (64%), Female -n=13 (36%).  RCT | Mean age of OCD sample =13.2, SD=3.1  Age range of OCD sample = 8.0-18.0 | CY-BOCS  SRS | Not reported on | Pearson’s correlation  Correlation of mean total SRS score and mean baseline total CY-BOCS score in total OCD sample r=0.087, p=0.064 | Not reported on | Data taken from RCT assessing an online Cognitive Bias Modification-Interpretation (CBMT-I) intervention designed to reduce dysfunctional interpration in youth with OCD symptoms  Limitation: Small sample size. Reliance on parent report for SRS. |
| Schachar et al.(2022)(24) | Canada  Total OCD clinic sample diagnosed according to DSM-V criteria- n=171. Male- n=102 (60%), Female – n=69(40%)  Case-control study | Mean age of clinic OCD sample=12.75,SD=2.55.  Age range of clinicl OCD sample =7-17.9. | - | 7.0% (n=12) of patients with a diagnosis of OCD, were also diagnosed with ASD (according to DSM-V).  75% of those diagnosed with ASD were male. | Not reported on | Not reported on | The main purpose of the study was to compare neurocognitive impairments between a clinic OCD and TD sample and a community OCD and TD sample.  Limitations – data could not be taken from the community OCD sample for the purposes of this meta-analysis as OCD diagnosis was parent reported and not verified. |
| Sevilla-Cermeno et al. (2019)(25) | Sweden  Total OCD child and adolescent sample according to ICD-10/ DSM-V criteria n=193. Male, n= 86 (44.6%), Female, n=107 (55.4%).  Total OCD sample including adults, n=31,856  Cohort Study | Mean age of OCD sample =13.6, SD-2.4  Age range of OCD sample =6-17 | CY-BOCS | 19.7% of patients aged 6-17 with a diagnosis of OCD also had a diagnosis of PDD according to ICD-10. | Not reported on | Not reported on | This study examined the prevalence of insomnia in individuals with OCD compared to the general population.  Limitations - patients on the National Patient Register (NPR) may not be representative of all patients. |
| Stewart el al. (2016)(26) | United State of America (USA)  Total OCD sample diagnosed according to DSM-IV-TR criteria- n=127  Male -n=60 (44.2%)  Female -n= 67 (55.8%)  No comparison group.  Cross-sectional but participants originally recruited as part of an RCT | Total OCD Sample Mean Age= 7.22, SD=1.2  Total OCD Sample Age Range=5-8. | SCQ  SRS  CY-BOCS | SRS t-score showed elevated autistic traits in this OCD sample - Mean=58.29, SD=11.21,n=127  36.2% of OCD sample scored >/= 60 on SRS.  ---------------------------------------  Mean SCQ score=5.74, SD=4.25.  2.4% of OCD sample scored >/=15 on SCQ | Pearson’s correlation used  CY-BOCS total t-score was positively correlated to the SRS total t-score - Pearson correlation – 0.21 p<0.05  CY-BOCS total t-score was positively correlated to the SRS subscale of autistic mannerisms t-score - Pearson correlation – 0.21 p<0.05  CY-BOCS total severity score did not significantly correlate with SCQ total score -Pearson Correlation – 0.11p>0.05  SCQ total and SRS total t-score did strongly significantly correlate Pearson correlation – 0.54 p<0.001 | Not reported on | Limitations - SRS may be capturing symptom overlap whilst the SCQ is not.  Milder spectrum of ASD traits may not be identified by the “yes/no” responses on the SCQ.  SRS may not be able to assess the function of the behaviour  Parents report on a topographical level of the symptoms they observe rather than on a functional level. |
| Sturm et al. 2018(27) | United States of America  Total OCD sample diagnosed according to DSM-IV criteria - n=32  Male -n = 15 (46.9)%  Female n=17-(53.1%)  Comparison -Tic Disorder group, ASD group and severe mood dysregulation group.  Case-control but participants originally recruited for intervention study | Total OCD Sample Mean Age =12.41, SD=3.03 | SRS | % of OCD group meeting clinical cut off scores (t>60) for total SRS score = 14.29  ---------------------------------------  % of OCD group meeting clinical cut off scores for social awareness subscale score = 18.75  ---------------------------------------  % of OCD group meeting clinical cut off scores for social cognition subscale score = 12.90  ---------------------------------------  % of OCD group meeting clinical cut off scores for social communication subscale score = 6.45  ---------------------------------------  % of OCD group meeting clinical cut off scores for social motivation subscale score = 19.35  ---------------------------------------  % of OCD group meeting clinical cut off scores for restricted and repetitive behaviour subscale score =22.58 | Not reported on | Not reported on | Limitations – no control group for comparison  Not necessarily generalisable to youth with more complex symptom presentations due to exclusion criteria.  Small sample size limits generalisability.  Findings relied on parental reporting. |
| Weidle et al.(2012)(28) | Sweden  Total OCD sample diagnosed according to DSM-IV criteria - n=105  Male -n =44 (41.9%)  Female - n= 61 (58.1%)  Age and gender matched control group from a Swedish town in the same geographical area-n=108  Case-control study | Total OCD Sample Mean Age = 13 | SCQ  CY-BOCS | SCQ total scores statistically significantly higher in the OCD group (m=5.9, SD-3.2, n=105) than in the control group (m=3.2, SD=3.1,n=108)  Independent t-test= 6.2250, df=211, p=0.0001, moderate effect size r=.40.  ---------------------------------------  SCQ>/= to 15 (score indicating likely ASD) – OCD group n=1 (0.95%) and Control group n=1 (0.94). OCD group.  SCQ>/= to 9 in OCD group = 21%, Fishers Exact Test, p=0.001 | Not reported on. | Not reported on | Study used a dimensional approach comparing current and pre-school ASD symptoms.  Limitations –  Some ASQ scores may be a result of faulty parental observations and interpretations.  Semi-structured interview such as ADIS or DISCO was not used. |
| Wolters et al.(2016)(29) | Denmark  Total OCD Sample - diagnosed according to DSM-IV TR criteria n=58  Male - n=24 (41.4%)  Female -n = 34 (58.6%)  No comparison group  Randomised Control Trial | Total OCD Sample Mean Age = 12.8, SD =2.6  Total OCD Sample Age Range =8-18 years. | CSBQ  CY-BOCS | CSBQ normoscore – mean =4.3 SD=1.6.  CSBQ normoscore range = 1-6  CSBQ normoscore >/= 95^th^ percentile n=16 | Baseline CBSQ and baseline CY-BOCS scores were significantly correlated r=.35, p=.009 | Not reported on | The main research question explored CBT efficacy related to OCD severity and ASD symptoms.  Limitations – the study was not designed to specifically assess ASD traits in an OCD sample. The study focussed on treatment outcomes.  There was no comparison control group to compare CSBQ scores to sample. |

ADHD – Attention Deficit Hyperactivity Disorder, AQ-- Autism Quotient, ASD- Autism Spectrum Disorder, ASQ -Autism Spectrum Quotient, ASSQ- Autism Spectrum Screening Questionnaire, CBT – Cognitive Behavioural Therapy, CGAS-Children’s Global Assessment Scale, CGI-S -Clinical Global Impression Severity Scale, COIS-P– Child Obsessive-Compulsive Impact Scale (parent version), COIS-R – Child Obsessive-Compulsive Impact Scale (Revised), CSBQ - Children’s Social Behaviour Questionnaire, CY-BOCS- Children’s Yale Brown Obsessive Compulsive Scale, DISCO- Diagnostic Interview for Social and Communication Disorders, DSM-IV-TR- Diagnostic and Statistical Manual of Mental Disorders (Fourth Revision), ICD-10 – International Classification of Diseases (Tenth Revision), IQ-Intelligence Quotient, IQR – Interquartile Range, m-mean, n-number, OCD- Obsessive Compulsive Disorder, OR- odds ration, NDT – Neurodevelopmental, p-probability, RCADS- Revised Children’s Anxiety and Depression Scale, RCT -Randomised Control Trial, RMET – Reading the Mind in the Eyes Test, ,SCQ- Social Communication Questionnaire, SD- Standard Deviation, SRS- Social Responsiveness Scale, SRS-2 - Social Responsiveness Scale–second edition.

References

1. Adam J, Goletz H, Mattausch SK, Plück J, Döpfner M. Psychometric evaluation of a parent-rating and self-rating inventory for pediatric obsessive-compulsive disorder: German OCD Inventory for Children and Adolescents (OCD-CA). Child Adolesc Psychiatry Ment Health. 2019;13:25.

2. Arildskov TW, Hojgaard DR, Skarphedinsson G, Thomsen PH, Ivarsson T, Weidle B, et al. Subclinical autism spectrum symptoms in pediatric obsessive-compulsive disorder. Eur Child Adolesc Psychiatry. 2016;25(7):711-23.

3. Boedhoe PSW, van Rooij D, Hoogman M, Twisk JWR, Schmaal L, Abe Y, et al. Subcortical Brain Volume, Regional Cortical Thickness, and Cortical Surface Area Across Disorders: Findings From the ENIGMA ADHD, ASD, and OCD Working Groups. Am J Psychiatry. 2020;177(9):834-43.

4. Chen MH, Tsai SJ, Liang CS, Cheng CM, Su TP, Chen TJ, et al. Diagnostic progression to schizophrenia in 35,255 patients with obsessive-compulsive disorder: a longitudinal follow-up study. Eur Arch Psychiatry Clin Neurosci. 2022.

5. Choi EJ, Vandewouw MM, Taylor MJ, Arnold PD, Brian J, Crosbie J, et al. Beyond diagnosis: Cross-diagnostic features in canonical resting-state networks in children with neurodevelopmental disorders. Neuroimage Clin. 2020;28:102476.

6. Farrell L, Waters A, Milliner E, Ollendick T. Comorbidity and treatment response in pediatric obsessive-compulsive disorder: A pilot study of group cognitive-behavioral treatment. Psychiatry research. 2012;199(2):115-23.

7. Geller DA, Biederman J, Griffin S, Jones J, Lefkowitz TR. Comorbidity of juvenile obsessive-compulsive disorder with disruptive behavior disorders. J Am Acad Child Adolesc Psychiatry. 1996;35(12):1637-46.

8. Griffiths DL, Farrell LJ, Waters AM, White SW. ASD Traits Among Youth with Obsessive-Compulsive Disorder. Child Psychiatry Hum Dev. 2017;48(6):911-21.

9. Griffiths DL, Farrell LJ, Waters AM, White SW. Clinical correlates of obsessive compulsive disorder and comorbid autism spectrum disorder in youth. Journal of Obsessive-Compulsive and Related Disorders. 2017;14:90-8.

10. Hanna GL. Demographic and clinical features of obsessive-compulsive disorder in children and adolescents. J Am Acad Child Adolesc Psychiatry. 1995;34(1):19-27.

11. Højgaard D, Arildskov TW, Skarphedinsson G, Hybel KA, Ivarsson T, Weidle B, et al. Do Autistic Traits Predict Outcome of Cognitive Behavioral Therapy in Pediatric Obsessive-Compulsive Disorder? Res Child Adolesc Psychopathol. 2023;51(8):1083-95.

12. Ivarsson T, Melin K. Autism spectrum traits in children and adolescents with obsessive-compulsive disorder (OCD). J Anxiety Disord. 2008;22(6):969-78.

13. Jaspers-Fayer F, Han SHJ, Chan E, McKenney K, Simpson A, Boyle A, et al. Prevalence of Acute-Onset Subtypes in Pediatric Obsessive-Compulsive Disorder. J Child Adolesc Psychopharmacol. 2017;27(4):332-41.

14. Jassi AD, Vidal-Ribas P, Krebs G, Mataix-Cols D, Monzani B. Examining clinical correlates, treatment outcomes and mediators in young people with comorbid obsessive-compulsive disorder and autism spectrum disorder. Eur Child Adolesc Psychiatry. 2021.

15. Lewin A, Wood J, Gunderson S, Murphy T, Storch E. Phenomenology of Comorbid Autism Spectrum and Obsessive-Compulsive Disorders Among Children. Journal of Developmental and Physical Disabilities - J DEV PHYS DISABILITIES. 2011;23.

16. Mahjoob M, Cardy R, Penner M, Anagnostou E, Andrade BF, Crosbie J, et al. Predictors of health-related quality of life for children with neurodevelopmental conditions. Sci Rep. 2024;14(1):6377.

17. Martin AF, Jassi A, Cullen AE, Broadbent M, Downs J, Krebs G. Co-occurring obsessive-compulsive disorder and autism spectrum disorder in young people: prevalence, clinical characteristics and outcomes. Eur Child Adolesc Psychiatry. 2020.

18. Oyku Memis C, Sevincok D, Dogan B, Baygin C, Ozbek M, Kutlu A, et al. The subthreshold autistic traits in patients with adult-onset obsessive-compulsive disorder: a comparative study with adolescent patients. Riv Psichiatr. 2019;54(4):168-74.

19. Onat M, Nas Unver AB, Senses Dinc G, Cop E, Pekcanlar Akay A. Comparisons between obsessive-compulsive disorder and trichotillomania in terms of autistic traits and repetitive behaviors in adolescents. Nordic Journal of Psychiatry. 2025;79(1):34-41.

20. Özyurt G, Beşiroğlu L. Autism Spectrum Symptoms in Children and Adolescents with Obsessive Compulsive Disorder and Their Mothers. Noro Psikiyatr Ars. 2018;55(1):40-8.

21. Peris TS, Rozenman M, Bergman RL, Chang S, O'Neill J, Piacentini J. Developmental and clinical predictors of comorbidity for youth with obsessive compulsive disorder. J Psychiatr Res. 2017;93:72-8.

22. Perez-Vigil A, Ilzarbe D, Garcia-Delgar B, Morer A, Pomares M, Puig O, et al. Theory of mind in neurodevelopmental disorders: Beyond autistic spectrum disorder. Neurologia. 2021.

23. Salemink E, Hagen A, de Haan E, Wolters L. Cognitive Bias Modification of Interpretation training for youth with OCD: Who benefits? Examining the role of OCD severity, interpretation bias, and autism symptoms. Journal of Obsessive-Compulsive and Related Disorders. 2023;37:100809.

24. Schachar RJ, Dupuis A, Anagnostou E, Georgiades S, Soreni N, Arnold PD, et al. Obsessive-compulsive disorder in children and youth: neurocognitive function in clinic and community samples. J Child Psychol Psychiatry. 2022;63(8):881-9.

25. Sevilla-Cermeño L, Isomura K, Larsson H, Åkerstedt T, Vilaplana-Pérez A, Lahera G, et al. Insomnia in obsessive-compulsive disorder: A Swedish population-based cohort study. J Affect Disord. 2020;266:413-6.

26. Stewart E, Cancilliere MK, Freeman J, Wellen B, Garcia A, Sapyta J, et al. Elevated Autism Spectrum Disorder Traits in Young Children with OCD. Child Psychiatry Hum Dev. 2016;47(6):993-1001.

27. Sturm A, Rozenman M, Chang S, McGough JJ, McCracken JT, Piacentini JC. Are the components of social reciprocity transdiagnostic across pediatric neurodevelopmental disorders? Evidence for common and disorder-specific social impairments. Psychiatry Res. 2018;264:119-23.

28. Weidle B, Melin K, Drotz E, Jozefiak T, Ivarsson T. Preschool and current autistic symptoms in children and adolescents with obsessive-compulsive disorder (OCD). Journal of Obsessive-Compulsive and Related Disorders. 2012;1(3):168-74.

29. Wolters LH, de Haan E, Hogendoorn SM, Boer F, Prins PJM. Severe pediatric obsessive compulsive disorder and co-morbid autistic symptoms: Effectiveness of cognitive behavioral therapy. Journal of Obsessive-Compulsive and Related Disorders. 2016;10:69-77.
